# Targeted demethylation of the BRD7 promoter based on CRISPR/dCas9 system inhibits the malignant progression of nasopharyngeal carcinoma

**DOI:** 10.1002/ctm2.70583

**Published:** 2026-01-09

**Authors:** Jianxia Wei, Yumei Duan, Changning Xue, Lemei Zheng, Qingqing Wei, Zubing Wu, Huizhen Xin, Ting Zeng, Hongyu Deng, Songqing Fan, Wei Xiong, Zhaoyang Zeng, Mengna Li, Ming Zhou

**Affiliations:** ^1^ NHC Key Laboratory of Carcinogenesis, Hunan Key Laboratory of Oncotarget Gene Hunan Cancer Hospital and the Affiliated Cancer Hospital of Xiangya School of Medicine, Central South University Changsha China; ^2^ Cancer Research Institute and Xiangya School of Basic Medical Sciences Central South University Changsha China; ^3^ The Key Laboratory of Carcinogenesis and Cancer Invasion of the Chinese Ministry of Education, Central South University Changsha China; ^4^ Department of Pathology the Xiangya Hospital, Central South University Changsha China; ^5^ Department of Pathology the Second Xiangya Hospital, Central South University Changsha China

**Keywords:** BRD7, CRISPR/dCas9, demethylation, malignant progression, nasopharyngeal carcinoma

## Abstract

**Background:**

BRD7 has been confirmed to be lowly expressed in nasopharyngeal carcinoma (NPC) tissues and exerts tumour suppressive roles. However, the molecular mechanism of the downregulation of BRD7 expression and whether the strategy of activating BRD7 expression plays anti‐tumour effects still needs to be clarified.

**Methods:**

Methylation‐specific polymerase chain reaction (PCR) was used to identify the methylation levels of BRD7 promoter. In vitro experiments were used to evaluate the effects of BRD7‐targeted demethylation system on the malignant progression of NPC cells. Chromatin immunoprecipitation (ChIP)‐qPCR experiment was employed to examine the regulatory mechanisms underlying the demethylation system. Xenograft tumour models were used to assess impact of this demethylation system on tumour growth in vivo and the anti‐tumour effects of the lentivirus‐mediated demethylation system in NPC.

**Results:**

There was hypermethylation modification in BRD7 promoter, which was negatively correlated with BRD7 expression. Next, we constructed a LentiCRISPRv2/dCas9‐TET1CD‐sgRNAs system targeting specific methylation sites of BRD7 promoter based on five sgRNAs, and confirmed that all five sgRNA‐guided CRISPR/dCas9 systems could activate BRD7 and inhibit cell proliferation to varying degrees, among which sgRNA2&sgRNA5 were the most significant. Further, we constructed NPC cell lines stably transfected with LentiCRISPRv2/dCas9‐TET1CD‐sgRNA2&5, and confirmed that both sgRNA2&sgRNA5 could promote the transcriptional activation by reducing its methylation, and inhibit the cell proliferation, migration, invasion and tumour growth in vivo of NPC, and the combination of them has a more significant demethylation, transcriptional activation and anti‐tumour effect. In addition, BRD7 had hypermethylation modification in its promoter and decreased expression in NPC tissues, and both of them were negatively correlated, making it a potential diagnostic marker for NPC diagnosis.

**Conclusions:**

The hypermethylation modification of BRD7 is an important mechanism leading to the inactivation of BRD7, and targeting demethylation of BRD7 inhibits the malignant progression of NPC, which might be a promising targeted therapeutic approach for treating NPC.

## Graphical abstract

1. Hypermethylation of BRD7 promoter CpG island is a critical mechanism leading to the downregulation of BRD7 expression in nasopharyngeal carcinoma (NPC).

2. LentiCRISPRv2/dCas9‐TET1CD‐sgRNAs demethylation system could promote transcriptional activation and expression of BRD7 by reducing its methylation.

3. LentiCRISPRv2/dCas9‐TET1CD‐sgRNAs demethylation system could inhibit tumour progression of NPC, and the combination of sgRNA2&sgRNA5‐guided demethylation system has a more significant demethylation, transcriptional activation and anti‐tumour effect.

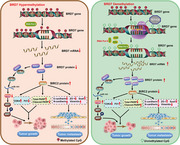

## 1 INTRODUCTION

Nasopharyngeal carcinoma (NPC) ranks as one of the most prevalent malignant tumours in southern China and seriously endangers human health.^1^, ^2^, ^3^ BRD7 was confirmed to be down‐expressed in NPC and acts as a tumour suppressor in NPC by negatively regulating signalling pathways like ERK, Rb/E2F and PI3K/AKT, thereby inhibiting tumour progression and metastasis.^4^, ^5^, ^6^, ^7^ In previous studies, we have confirmed that BRD7 is a highly unstable protein in breast cancer, and TRIM28‐mediated BRD7 instability is a significant mechanism contributing to the onset and development of breast cancer.^8^ Additionally, we found that BRD7 was lowly expressed at the transcriptional levels in NPC and breast cancer, and it was negatively regulated by c‐Myc, resulting in its downregulation at the transcriptional level.^9^ This suggests that the low expression at the transcriptional level is a significant mechanism contributing to the occurrence and progression of NPC.

There are many mechanisms underlying low mRNA expression, among which methylation modifications are a critical epigenetic modification, which lead to abnormal gene expression and the occurrence of disease.^10^, ^11^, ^12^ Studies have shown that cytosine bases in the cytosine‐guanine (CpG) islands of tumour suppressor gene promoters are often methylated to form 5‐methylcytosine (5mC). This type of abnormal hypermethylation may result in transcriptional silencing, consequently facilitating tumourigenesis and progression.^13^, ^14^ Several genes, including p16, Rb, PTEN, ZNF154 and BRCA1, have been identified as hypermethylated in corresponding tumours.^15^, ^16^, ^17^, ^18^, ^19^, ^20^ In this study, we found that there was hypermethylation modifications in the BRD7 promoter through bioinformatics prediction, suggesting that hypermethylation might be an important mechanism leading to the downregulation of BRD7 expression.

Studies have shown that the methylation process is dynamic, and demethylase can remove 5mC in order to revert to the unmethylated condition.^21^, ^22^ Therefore, inhibiting the hypermethylation of tumour suppressor genes is a promising strategy for tumour treatment. Azacitidine (AZA) and decitabine (5‐Aza‐2′‐deoxycytidine, 5‐AZA‐CdR) are two broad‐spectrum DNA methyltransferase inhibitors (DNMTis) authorised by the US Food and Drug Administration (FDA).^23^ As DNA demethylating drugs, they have been shown to be effective in treating myelodysplastic syndromes (MDS), leukaemia and other malignancies.^24^, ^25^, ^26^ However, the global demethylation induced by these broad‐spectrum demethylating drugs has undesirable side effects, and their lack of specificity limits their clinical application.^27^ Therefore, there is an urgent need to discover new generations of targeted DNA demethylation methods that are both highly potent and highly selective. To attain more accurate DNA demethylation, some researchers engineered the CRISPR/dCas9 system, leveraging the ease and specificity of the CRISPR/Cas9 system, which has been utilised for precise epigenomic modifications by linking to epigenomic regulatory elements.^28^, ^29^, ^30^ TET family are key participants in DNA demethylation, and of them, TET1 possesses a TET catalytic domain, which is essential in the demethylation process.^31^ The CRISPR/dCas9‐TET1CD‐sgRNAs system is composed of a nuclease‐deficient Cas9 (dCas9) and TET1 catalytic domain (TET1CD), which has been reported to specifically and effectively regulate the methylation modifications of target genes under the guidance of sgRNAs, without affecting other genes, thereby facilitating gene regulation.^19^, ^32^, ^33^, ^34^

In this study, we constructed a LentiCRISPRv2/dCas9‐TET1CD‐sgRNAs system targeting specific methylation sites of BRD7 promoter region based on five sgRNAs (sgRNA1‐5), and confirmed that sgRNA2 and sgRNA5‐guided dCas9‐TET1CD system could dramatically reduce methylation level and promote the transcriptional activation and BRD7 expression, thereby inhibiting tumour malignant progression and play anti‐tumour effects in NPC, and the combination of the two sgRNAs exhibited a more pronounced effects. Additionally, hypermethylation was found in CpG island region of BRD7 promoter in NPC clinical specimens, which displayed a negative correlation with BRD7 expression and a positive correlation with clinical stage. These results indicate that hypermethylation is an important molecular mechanism underlying the inactivation of BRD7 expression and tumour progression in NPC, and targeting BRD7 demethylation can effectively inhibit the malignant progression of NPC, thus offering a potential treatment strategy for NPC patients.

## 2 METHODS

### 2.1 Clinical tissue samples

All clinical tissues specimens were obtained from the Second Xiangya Hospital. All experimental tissue samples in the study were obtained with the approval by the Ethics Committee of Central South University and the written informed consent of patients.

### 2.2 Cell lines and cell culture

Human NPC cell lines were cultured in Dulbecco's modified Eagle medium (DMEM) (Cat No. 11960051, Life Technologies) enriched with 10% foetal bovine serum (FBS; Cat No. 3022A, Umedium). The nasopharyngeal epithelial (NPE) cell line NP69 was propagated in Defined Keratinocyte‐SFM (Cat No. 10744019, 1×, Gibco). All cell lines were kept at 37°C with 5% CO_2_.

### 2.3 RNA extraction and quantitative RT‐PCR

The quantitative reverse transcription polymerase chain reaction (qRT‐PCR) analysis was conducted as outlined in previous study.^8^ Briefly, RNA was isolated utilising Trizol (Cat No. AG21102, Accurate Biology). Reverse transcription was carried out to generate cDNA by using the reverse transcription kit (Cat No. K1691, Thermo Scientific). The qPCR protocol used the qPCR Kit (Cat No. QP101A, YoungGen) following the manufacturer's guidelines.

### 2.4 Western blot

The methods of Western blot experiments are as previously published.^35^, ^36^ Specifically, Western and IP lysis buffer containing 1% protease inhibitor was used to extract protein. Furthermore, 25–40 µg protein was isolated and transferred to polyvinylidene fluoride (PVDF) membrane. The membrane was sealed with 5% skim milk for 1 h, followed by incubation at 4°C overnight with antibodies against then combined with BRD7 (Cat No. 51009‐2‐AP, Proteintech), ZO‐1 (Cat No. 21773‐1‐AP, Proteintech), E‐cadherin (Cat No. A20798, Abclonal), N‐cadherin (Cat No. AF5239, Affinity), Vimentin (Cat No. 10366‐1‐AP, Proteintech), Snail (Cat No. A5243, Abclonal), Total‐PARP (Cat No. 13371‐1‐AP, Proteintech), Cleaved‐PARP (Cat No. AF7023, Affinity), CDK4 (Cat No. 11026‐1‐AP, Proteintech), P21 (Cat No. 2947, CST) and Glyceraldehyde 3‐phosphate dehydrogenase (GAPDH) (Cat No. 10494‐1‐AP, Proteintech). On the second day, secondary antibodies labelled with horseradish peroxidase (Cat No. S0001, Affinity) were incubated for 1 h and visualised using a SuperKine™ Universal ECL Substrate kit (Cat No. BMP3010, Abbkine).

### 2.5 Methylation‐specific PCR

The extraction of genomic DNA from NPC cell lines and NPE cell line NP69 was executed with specific kits (Cat No. 51304, QIAGEN) following the manufacturer's guidelines. EpiArt DNA Methylation Bisulfite Kit (Cat No. EM101‐01, Vazyme) was utilised for bisulphite conversion. The DNA that underwent bisulphite treatment was PCR amplified with two sets of primers, which are detailed in Table S1. EpiArt HS Taq DNA Polymerase (Cat No. EM201‐01, Vazyme) was used to amplify the target genes. Following electrophoresis, the results were captured using a gel imaging system.

### 2.6 Decitabine (5‐Aza‐2′‐deoxycytidine) treatment

Briefly, CNE2 and 5‐8F cells were plated in six‐well plates. Upon reaching a confluency of 70%–80%, they were exposed to 10 µM 5‐AZA‐CdR (Cat No. A1906, APEXBIO) for a duration of 72 h. The drug‐containing medium was changed every 24 h. RNA was extracted, and following semi‐quantitative RT‐PCR was conducted as previously described.

### 2.7 SgRNA target design and off‐target identification

The sgRNA sequences targeting the highly methylated proximal promoter region of BRD7 (−404/+46) were designed using the CRISPick website. Subsequently, we selected the sgRNAs with an On‐Target Efficacy Score greater than .8 and only one recognition site in the target region. The five sgRNAs that met the screening criteria were named sgRNA1‐5, respectively. The sequences, efficiency scores, the direction of the targeted DNA double strand and target gene regions targeted by the sgRNAs are detailed in Table S2. Additionally, we performed a genome‐wide off‐target analysis on the two sgRNAs we ultimately selected (sgRNA2&sgRNA5) utilising the Cas‐OFFinder tool (http://www.rgenome.net/cas‐offinder/). We set the search criteria to allow for fewer than 3 base mismatches with the corresponding sgRNA sequences, focusing on potential off‐target sites in coding regions or promoter regions. The analysis results indicated that the sgRNAs we selected did not predict any off‐target sites with high homology.

### 2.8 The construction of the dCas9‐based demethylation plasmid and the relevant stable cell lines

First, we double‐cut the LentiCRISPRv2 vector with restriction enzymes to remove the Cas9 element and expose the cleavage site. The dCas9 domain was cloned from the dCas9‐VP64_GFP plasmid (YouBio, VT8131), and the TET1CD was obtained from the Addgene plasmid repository (https://www.addgene.org/). The fusion protein of TET1CD and dCas9 is assembled into the LentiCRISPRv2‐△Cas9 plasmid by standard restriction endonuclease enzyme digestion and ligation method. Thus, we obtained the demethylated plasmid LentiCRISPRv2/dCas9‐TET1CD. Subsequently, we connected five sgRNAs specifically targeting the hypermethylated region of the CpG island of the BRD7 promoter into the plasmid LentiCRISPRv2/dCas9‐TET1CD (sgRNAs sequences are shown in Table S2), and Sanger sequencing was correct. Then, the optimal combination of sgRNAs and dCas9‐TET1CD was selected to construct stable cell lines. Specifically, 293T cells were transfected with demethylated plasmids and the packaging plasmids pMD2.G and psPAX2. After transfection for 72 h, the lentiviral supernatant was collected, filtered and concentrated to infect NPC cells. After that, it was screened with purinomycin (Cat No. S250J0, BasslMedia, 1 µg/mL).

### 2.9 CCK‐8 assay

For each cell line, 1000 cells in 100 µL culture medium per well were plated in a 96‐well plate. Ten microlitre CCK‐8 reagent (Cat No. B34304, Selleck) was introduced and incubated at 37°C for 2–4 h at specified designated time intervals (0, 24, 48, 72, 96 and 120 h).

### 2.10 Colony formation assay

Cells were plated in six‐well plates at a density of 1000 cells per well for 14 days. Afterwards, cells were subjected to phosphate‐buffered saline (PBS) washing, fixation using 4% formaldehyde and staining by crystal violet. The colonies in each well were then counted.

### 2.11 Wound healing assay

Briefly, cells were plated in six‐well plates to full confluency. A linear scratch was then created in the monolayer using a sterile 10 µL pipette tip, and the cells were subsequently maintained in serum‐free medium. Wound closure was monitored by capturing images at 0, 24 and 48 h post‐scratch.

### 2.12 Transwell invasion assay

A 20 µL 1:8 Matrigel/serum‐free medium mixture was placed in the upper chamber and incubated for 3 h. DMEM or Roswell Park Memorial Institute (RPMI) 1640 culture medium supplemented with 20% FBS was introduced into the lower well. 5 × 10^4^ cells suspended in 200 µL serum‐free medium were introduced into upper chamber. Following a 48 h incubation period, the cells were subjected to fixation in 4% formaldehyde, and staining was performed using crystal violet.

### 2.13 Chromatin immunoprecipitation assays

Chromatin immunoprecipitation (ChIP) was conducted following our previously published protocol.^37^, ^38^ In brief, cells were cross‐linked, after which they were collected and lysed. The chromatin was decomposed into 100–500 bp fragments using the BioRuptor ultrasound instrument (Diagenode) by adding a nucleolytic buffer to the precipitation. Following the manufacturer's instructions, chromatin immunoprecipitation was performed with immunoglobulin IgG (Sigma) and anti‐5mC antibodies (Cat No. RM231, Abcam) or RNA Pol II (Cat No. A21980, Abclonal) and Protein A/G magnetic bead system (Cat No. B23202, Selleck Chemicals). Immunoprecipitation was performed overnight at 4°C, and after treatment with RNase A (Cat No. 12091021, Life Technologies), DNA–protein complexes were extracted using protease K, and the DNA was then purified. This DNA was then applied to RT‐qPCR. The corresponding ChIP‐qPCR primer sequences are shown in Table S3.

### 2.14 In vivo assays

Female BALB/C nude mice (4 weeks old) were obtained from Hunan Slyke Jingda Experimental Animal Co., Ltd. All mice were raised within the SPF barrier system at Central South University's Laboratory Animal Science Department. To establish xenograft tumour model, the mice were randomly assigned to four groups (five mice per group), including CNE2/dCas9‐TET1CD‐sgNC, CNE2/dCas9‐TET1CD‐sg2, CNE2/dCas9‐TET1CD‐sg5 and CNE2/dCas9‐TET1CD‐sg2+5. A subcutaneous tumour model was established by injecting 3 × 10^6^ CNE2 cells into the right flank of every nude mouse. Tumour size was assessed every 3 days, with volume determined by the formula: volume = (length × width^2^)/2. Following a 30‐day period, all animals were euthanised. The excised tumour masses were then weighed, fixed in 4% paraformaldehyde and embedded in paraffin blocks.

To establish lentivirus‐treated NPC xenograft model, 4 × 10^6^ CNE2 cells were inoculated into the right subcutaneous position of nude mice. These mice were then randomly divided into five groups (PBS group, LV‐dCas9‐TET1CD‐sgNC group, LV‐dCas9‐TET1CD‐sg2 group, LV‐dCas9‐TET1CD‐sg5 group and LV‐dCas9‐TET1CD‐sg2+5 group), with seven mice per group. When the tumour volume reached 40–50 mm^3^, 100 µL of lentivirus stably expressing dCas9‐TET1CD‐sgRNA2, dCas9‐TET1CD‐sgRNA5 and combination of them with a concentration of 1 × 10^8^ TU/mL were intratumourally injected, respectively, with a total of three times and once every 4 days. The negative control group received an equivalent volume containing the LV‐dCas9‐TET1CD‐sgRNANC virus, and the blank control group was administered an equivalent volume of PBS. The growth of the mouse xenografts was monitored, with tumour measurements taken every 2 days. On day 18 of the tumour inhibition study, the animals were euthanised. Subcutaneous tumour tissues were carefully dissected and weighed. The tumour inhibition rate (%) = [(Mean tumour volume of PBS group − Mean tumour volume of lentivirus treatment group or negative control group)/Mean tumour volume of PBS group] × 100%. A portion of tumour masses was fixed into 4% paraformaldehyde and embedded in paraffin blocks for future use, while another portion was stored at −80°C.

### 2.15 Immunohistochemical staining

The immunohistochemical (IHC) assay was conducted as previously outlined.^36^ Briefly, tumour tissue sections were deparaffinised, rehydrated and antigen retrieved. Following this, the sections were treated with primary antibodies targeting BRD7 (1:400, Cat No. 51009‐2‐AP, Proteintech), Ki67 (1:400, Cat No. BS40169, Bioworld), CDK4 (1:400, Cat No. 11026‐1‐AP, Proteintech), P21 (1:400, Cat No. 10355‐1‐AP, Proteintech), Vimentin (1:400, Cat No. 10366‐1‐AP, Proteintech), Cleaved‐PARP (1:400, Cat No. AF7023, Affinity) and ZO‐1 (1:1000, Cat No. 21773‐1‐AP, Proteintech) in a moist dark chamber overnight at 4°C. On the subsequent day, sections were treated with secondary antibody for 1 h. DAB was utilised to develop the staining, and nuclear counterstaining was performed with haematoxylin (Cat No. 517‐28‐2, Solarbio). The IHC results were evaluated based on the intensity of staining and distribution of the positive cells, and specific scoring criteria have been published.^36^, ^38^, ^39^, ^40^

### 2.16 Statistical analysis

Data were analysed using GraphPad Prism 7.0 software. All data were presented as mean ± standard error of the mean (SEM). Student's *t*‐test was used to employed any two groups. One‐way analysis of variance (ANOVA) was used for multiple. *p* values below .05 were deemed statistically significant (ns, no significance; *p < .05; **p < .01; ***p < .001).

## 3 RESULTS

### 3.1 BRD7 is down‐regulated in nasopharyngeal carcinoma

**FIGURE 1:**
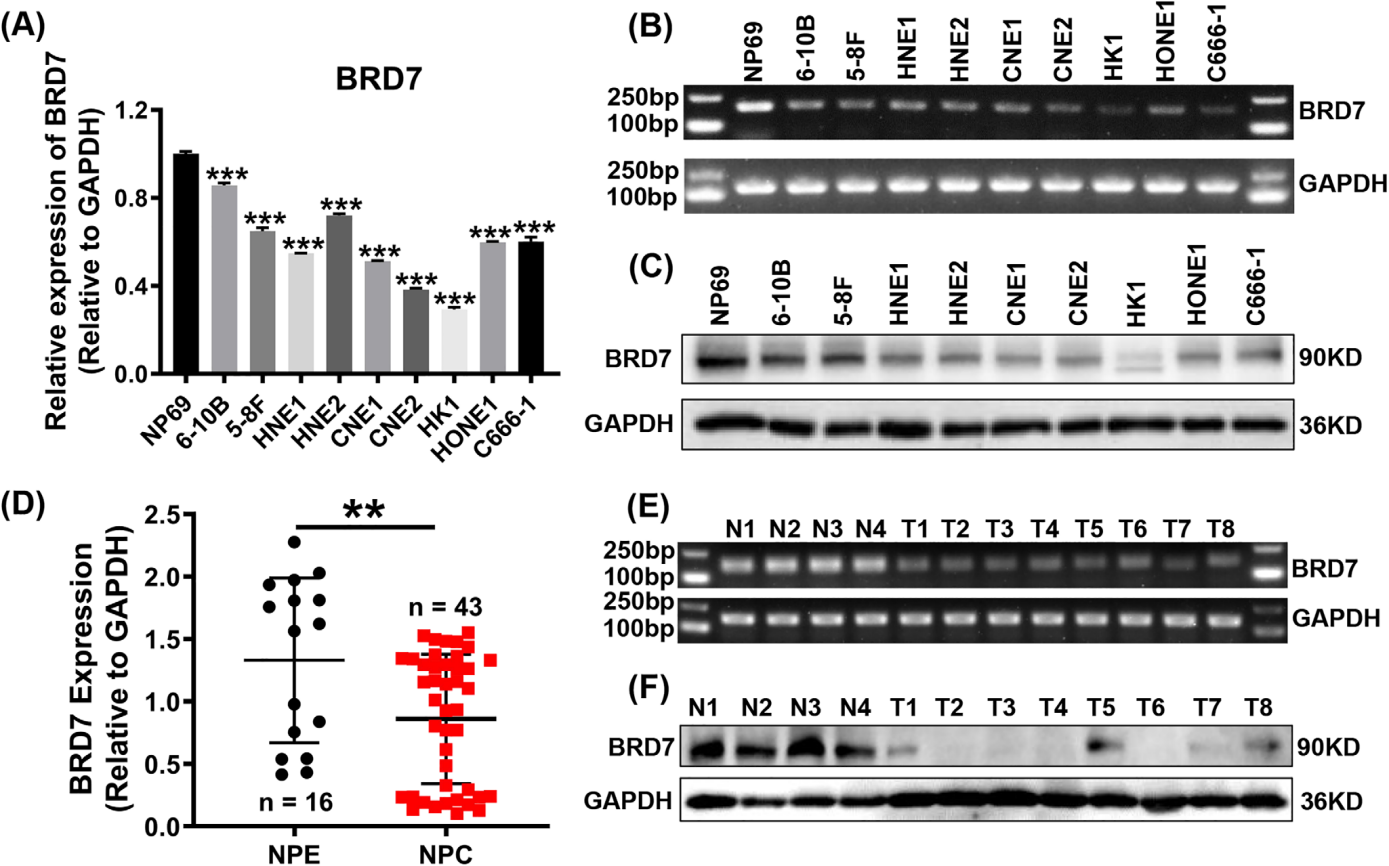
BRD7 is down‐regulated in nasopharyngeal carcinoma. (A–C) Reverse transcription‐quantitative polymerase chain reaction (RT‐qPCR) (A), Agarose gel electrophoresis (B) and Western blot (C) assays were used to detect BRD7 expression in nasopharyngeal epithelial (NPE) cell NP69 and nine nasopharyngeal carcinoma (NPC) cell lines (6‐10B, 5‐8F, HNE1, HNE2, CNE1, CNE2, HK1, HONE1 and C666‐1). The data are presented as mean ± standard error of the mean (SEM) (*n* = 3). (D–F) RT‐qPCR (D), Agarose gel electrophoresis (E) and Western blot (F) assays were used to detect BRD7 expression in 16 non‐cancerous nasopharyngeal tissues and 43 biopsy tissues of NPC. The sizes of the agarose gel electrophoresis products for BRD7 and GAPDH are 191 and 158 bp, respectively. The data are presented as mean ± SEM. **p < .01; ***p < .001.

Firstly, the expression of BRD7 in normal NPE cells and nine NPC cell lines was assessed using RT‐PCR and Western blot assays. The findings confirmed that the BRD7 expression level in NPC cell lines was lower compared to that in normal NPE cell line NP69 (Figure 1A–C). To further investigate the clinical significance of BRD7 in NPC clinical samples, we further collected fresh tissue samples from 16 non‐cancerous nasopharyngeal (NP) patients and 43 NPC patients. RT‐PCR and Western blot analyses demonstrated that BRD7 expression in NPC patients samples was markedly lower than in non‐cancerous NP tissues (Figure 1D–F), confirming the downregulation of BRD7 in NPC.

### 3.2 Hypermethylation of the CpG island in the BRD7 promoter mediates the downregulation of BRD7 expression in NPC

**FIGURE 2:**
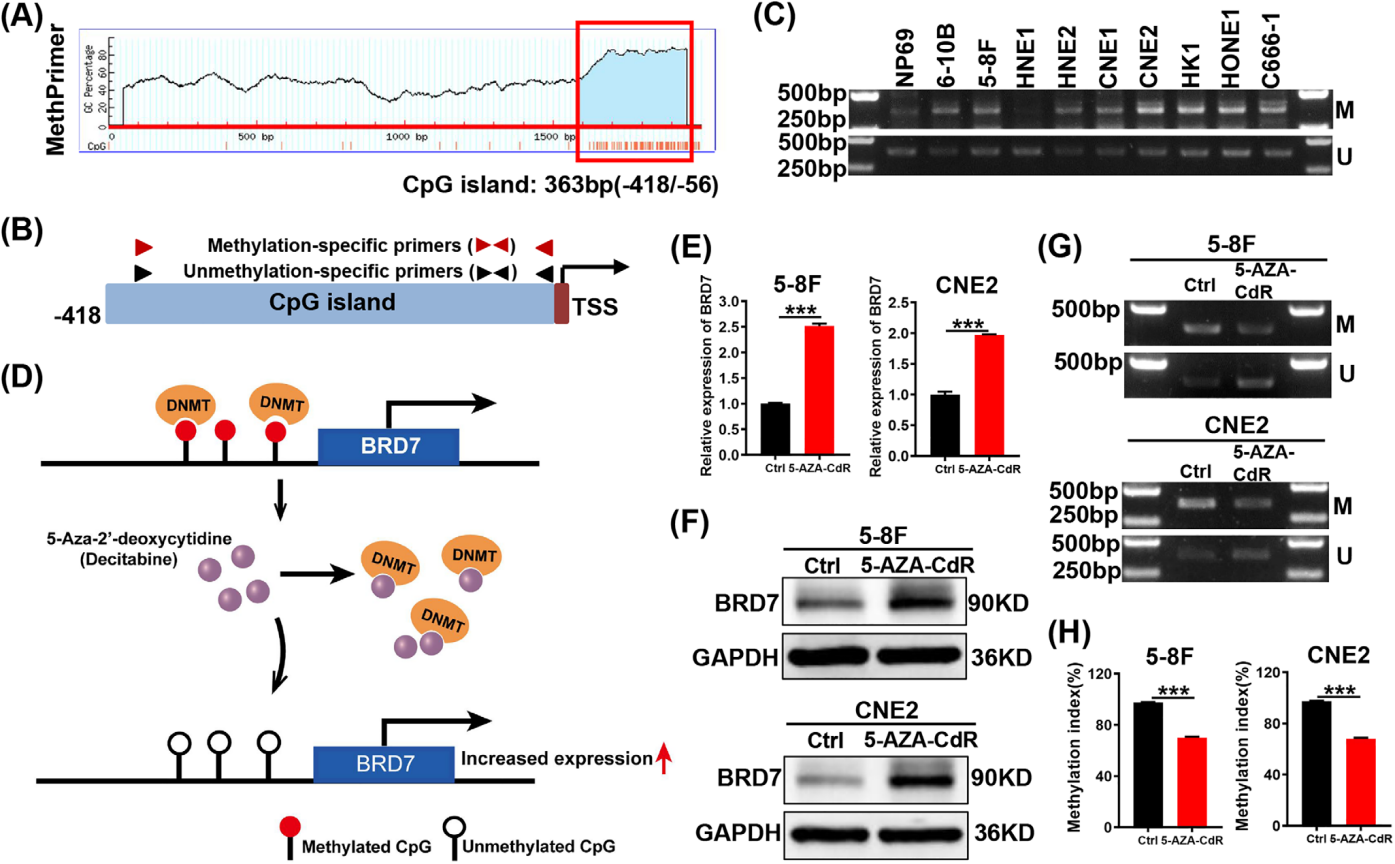
Hypermethylation of the CpG island in the BRD7 promoter mediates the downregulation of BRD7 expression in nasopharyngeal carcinoma (NPC). (A) Prediction of the CpG island region within the BRD7 promoter using the Methprimer website. (B) Schematic design of methylation‐specific polymerase chain reaction (PCR) primers. (C) The methylation state of BRD7 promoter in nasopharyngeal epithelial (NPE) cell NP69 and nine NPC cell lines (6‐10B, 5‐8F, HNE1, HNE2, CNE1, CNE2, HK1, HONE1 and C666‐1) was detected by methylation‐specific PCR (MSP). (D) Schematic diagram of demethylation of decitabine. DNMT, DNA methyltransferase. (E–G) Changes of BRD7 mRNA expression (E), protein expression (F) and methylation levels (G) in the two NPC cell lines before and after 5‐AZA‐CdR treatment for 3 days. M, methylated product (product size 379 bp); U, unmethylated product (product size 377 bp). (H) The qMSP experiment was conducted to detect the effects of decitabine treatment on the methylation levels of the CpG island region in the BRD7 promoter of NPC cells. In E and H, the data are presented as mean ± standard error of the mean (SEM) (*n* = 3). ***p < .001.

To investigate the mechanism of downregulation of BRD7 expression in NPC, we conducted bioinformatics analysis using publicly available database MethPrimer website to predict the size and location of the CpG island in BRD7 promoter region. The results revealed that the CpG island is located 56–418 bp upstream of the BRD7 transcription start site (−418/−56; Figure 2A), overlapping with the proximal promoter region of BRD7 (−404/+46). To further validate the methylation status of the BRD7 promoter, we treated genomic DNA from NPE cell NP69 and nine NPC cell lines with bisulphite. After treatment, all unmethylated cytosines are transformed into uracil, while methylated cytosines remain unchanged. Methylation‐specific PCR (MSP) amplification was subsequently carried out using two pairs of methylation‐specific and unmethylation‐specific primers. The positions of the primers are shown in Figure 2B, and their sequences can be found in Table S1. The results of the MSP confirmed that the BRD7 promoter exhibited hypermethylation of the CpG island in NPC cell lines compared to NPE cell NP69 (Figure 2C). Based on these findings, we hypothesise that hypermethylation of the BRD7 promoter may play a crucial role in NPC development by silencing BRD7 expression. In addition, NPC cell lines treated with Decitabine, a strong inhibitor of DNA methyltransferase, with the mechanism of action shown in Figure 2D, exhibited a notable increase in BRD7 mRNA and protein expression levels (Figure 2E,F), along with a significant reduction in the methylation level of the BRD7 promoter and an increase in the unmethylated level (Figure 2G). The qMSP experiment also confirmed the reduction in methylation levels of the BRD7 promoter region following decitabine treatment. However, we also found that this inhibitory effect is very weak, indicating its lack of target specificity (Figure 2H). These results clearly indicate that hypermethylation of the BRD7 promoter is a key mechanism underlying the downregulation of BRD7 expression.

### 3.3 The LentiCRISPRv2/dCas9‐TET1CD‐sgRNAs system targets demethylation of BRD7 in NPC cells

**FIGURE 3:**
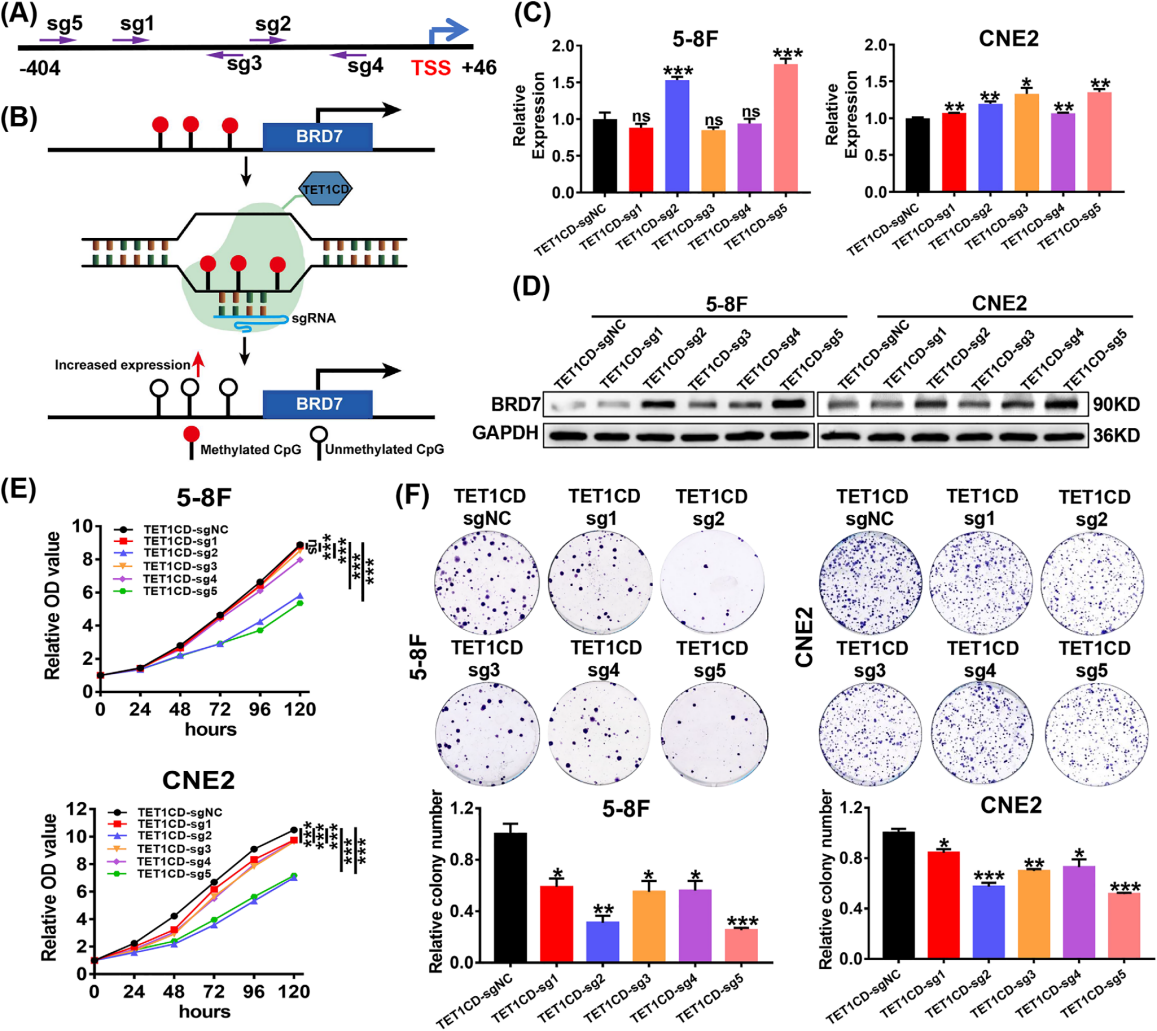
The LentiCRISPRv2/dCas9‐TET1CD‐sgRNAs system targets demethylation of BRD7 in nasopharyngeal carcinoma (NPC) cells. (A) Five sgRNAs were designed to target the hypermethylated region of the BRD7 promoter CpG island (indicated by the arrow). TSS, transcription start site. (B) Schematic diagram of the LentiCRISPRV2/dCas9‐TET1CD‐sgRNAs demethylation system targeting demethylated BRD7. (C, D) BRD7 mRNA (C) and protein (D) expression levels in NPC cells were determined after transfection with demethylation system. In C, the data are presented as mean ± standard error of the mean (SEM) (*n* = 3). (E, F) CCK‐8 assay (*n* = 5, five replicates per group) (E) and colony formation assay (*n* = 3, three replicates per group) (F) were applied to detect cell viability and the ability of colony formation of NPC cells after transfection of dCas9‐TET1CD‐sgNC and dCas9‐TET1CD‐sgRNAs (sgRNA1–5). In C, E and F, the data are presented as mean ± SEM. *p < .05; **p < .01; ***p < .001; ns, no significance.

To activate BRD7 expression through specific demethylation, we constructed a LentiCRISPRv2/dCas9‐TET1CD‐sgRNAs specific demethylation system based on the dCas9‐guided demethylation technique (Figure S1A), and five specific sgRNAs targeting the hypermethylated areas of the CpG island within the BRD7 promoter were designed (Figure 3A), and then respectively inserted into the construct, and Sanger sequencing confirmed that the demethylation system was successfully constructed (Figure S1B). Theoretically, the sgRNAs in this system could recruit the dCas9‐TET1CD fusion protein to the hypermethylated regions of the BRD7 promoter CpG island, thereby reducing the DNA methylation of BRD7 promoter region (Figure 3B). To confirm the impact of this system, we transiently transfected the LentiCRISPRv2/dCas9‐TET1CD‐sgRNAs (sgRNA1‐5) system into BRD7 low‐expressing NPC cell lines 5‐8F and CNE2, and the results of RT‐qPCR and Western blot assays confirmed that the five sgRNAs‐guided dCas9‐TET1CD system could promote BRD7 expression at mRNA and protein levels in NPC cells to varying degrees when compared to the control group, of which the sgRNA2 and sgRNA5 present the most pronounced effects (Figure 3C,D). Additionally, CCK‐8 and colony formation analyses also verified that under the guidance of sgRNA2 and sgRNA5, the system effectively suppressed the proliferation ability and colony formation ability of NPC cells 5‐8F and CNE2, with the most significant inhibitory effects (Figure 3E,F). In addition, we additionally included two control groups, dCas9 and dCas9/TET1CD‐scrambled sgRNA, and further confirmed through Western blot and CCK‐8 experiments that under the guidance of sgRNA2 and sgRNA5, the dCas9‐TET1CD demethylation system can specifically and significantly activate BRD7 expression, thereby inhibiting the proliferation of NPC cells (Figure S2). Therefore, we selected these two optimal sgRNAs‐guided CRISPR/dCas9 systems for the subsequent studies on anti‐tumour utilisation and mechanisms.

### 3.4 Targeted demethylation of the BRD7 promoter activates the expression of BRD7

**FIGURE 4:**
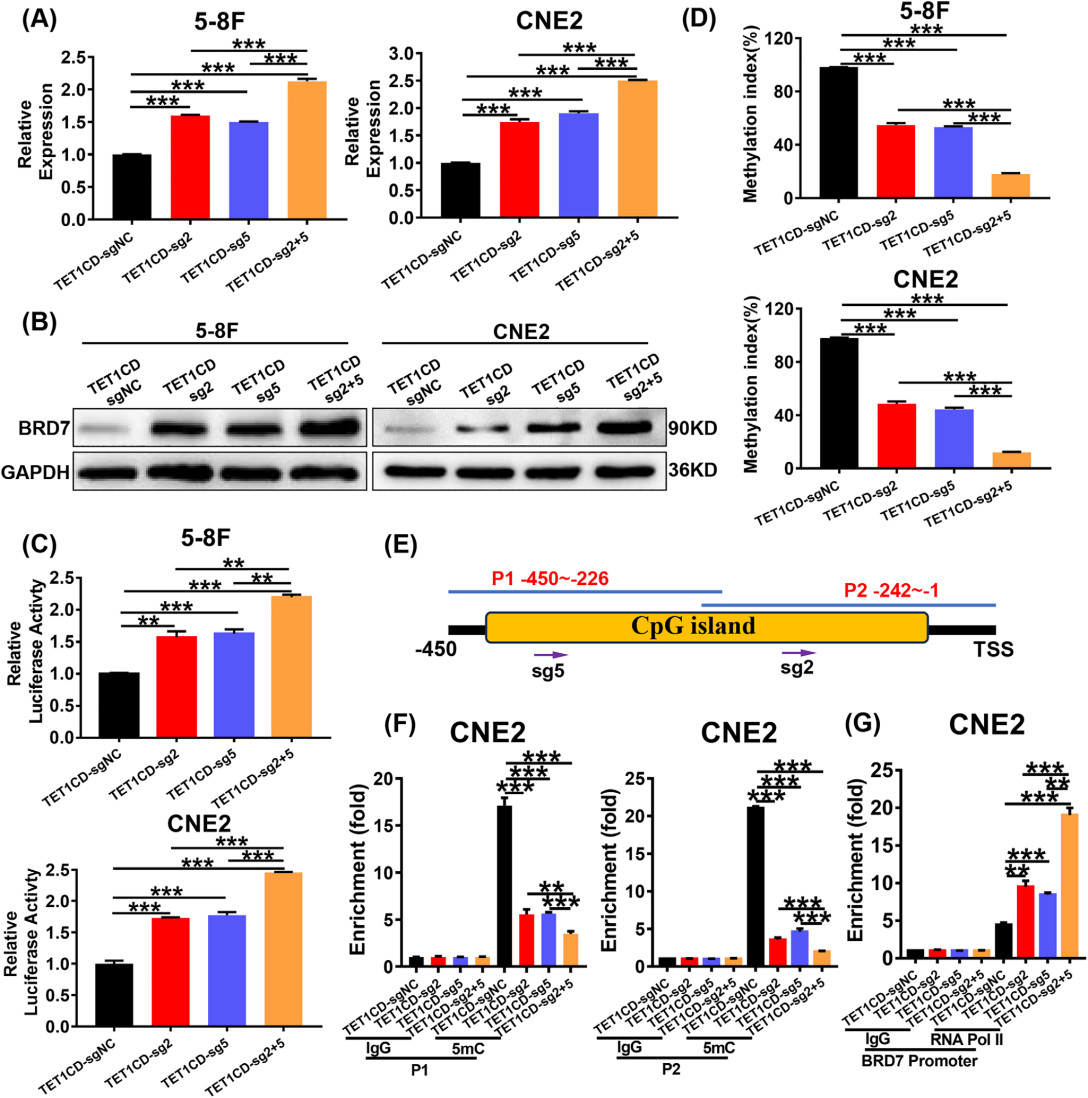
Targeted demethylation of the BRD7 promoter activates the expression of BRD7. (A, B) BRD7 mRNA levels (A) and protein levels (B) in nasopharyngeal carcinoma (NPC) cells stably expressing dCas9‐TET1CD‐sgNC, dCas9‐TET1CD‐sgRNA2, dCas9‐TET1CD‐sgRNA5 and co‐expressing dCas9‐TET1CD‐sgRNA2 and dCas9‐TET1CD‐sgRNA5. (C) Dual‐luciferase assay was used to detect the effects of sgRNA2 or sgRNA5 paired alone with dCas9‐TET1CD and the combined delivery of sgRNA2 and sgRNA5 with dCas9‐TET1CD on the activity of the BRD7 promoter reporter plasmid luciferase. (D) The qMSP experiment was conducted to assess the effects of the dCas9‐TET1CD‐sgRNAs demethylation system treatment on the methylation levels of the CpG island region in the BRD7 promoter of NPC cells. (E) The primers’ positions in chromatin immunoprecipitation‐quantitative polymerase chain reaction (ChIP‐qPCR). (F, G) ChIP‐qPCR experiments were conducted to assess the effect of this system on the methylation levels (F) and RNA Pol II levels (G) of BRD7 promoter region. In A, C, D, F and G, the data are presented as mean ± standard error of the mean (SEM) (*n* = 3). **p < .01; ***p < .001.

To further confirm the mechanism by which the sgRNAs‐guided CRISPR/dCas9 systems activate the expression of BRD7, we established stable cell lines expressing dCas9‐TET1CD‐sgRNA2 and dCas9‐TET1CD‐sgRNA5 by infecting NPC cells with the lentiviral supernatant. RT‐qPCR and Western blot analyses indicated that, in comparison to the control group, the mRNA and protein levels of BRD7 were significantly increased in NPC cells stably expressing dCas9‐TET1CD‐sgRNA2 or dCas9‐TET1CD‐sgRNA5 (Figure 4A,B).

As previous studies have shown that co‐delivery of multiple sgRNAs can increase endogenous genes activation levels, therefore, we further transfected the plasmid LentiCRISPRv2/dCas9‐TET1CD‐sgRNA5 into NPC cells 5‐8F and CNE2 that stably expressed dCas9‐TET1CD‐sgRNA2 to assess the activation effect of the combination of these two sgRNAs on BRD7 expression. RT‐qPCR and Western blot experiments indicated that, compared to cells stably expressing either dCas9‐TET1CD‐sgRNA2 or dCas9‐TET1CD‐sgRNA5 alone, the combined delivery of sgRNA2 and sgRNA5 exhibited an additive effect, leading to a more significant activation of BRD7 (Figure 4A,B). Immunofluorescence experiments also confirmed the activation of BRD7 mediated by the demethylation system. It was also found that this system did not affect the localization of BRD7, which remained primarily concentrated in the nucleus, indicating that the activation of BRD7 mediated by this demethylation system may be achieved by regulating BRD7's activity within the nucleus (Figure S3). Dual‐luciferase assay results also confirmed that stable expression of this system could enhance luciferase activity of the BRD7 proximal promoter, and the combined delivery of the two sgRNAs presented more dramatic activation effect (Figure 4C). Meanwhile, the qMSP results indicate that in NPC cells, stable expression of dCas9‐TET1CD‐sgRNA2&sgRNA5 significantly reduced methylation levels in BRD7 promoter CpG island when compared to the control group. Furthermore, the combined delivery of sgRNA2 and sgRNA5 showed a synergistic effect, resulting in even more pronounced inhibition. This confirms that our demethylation system can activate BRD7 expression by specifically targeting and demethylating the highly methylated regions of the BRD7 promoter (Figure 4D). Subsequently, we designed primers (P1 and P2) targeting the regions where sgRNA2 and sgRNA5 are located, with their locations shown in Figure 4E. ChIP‐qPCR assays were performed to assess the impact of this system on the methylation levels within the BRD7 promoter region. The results showed that the CpG island region was highly methylated in 5‐8F and CNE2 cells, while sgRNA2 and sgRNA5‐guided dCas9‐TET1CD significantly inhibited the methylation levels, with the combined delivery showing the most pronounced inhibitory effect on methylation levels (Figure 4F). At the same time, this demethylation system was found to have a more significant inhibitory effect on the methylation level of the −242/−1 segment (Figure 4F). Given the critical role of RNA polymerase II (RNA Pol II) in transcription activation, we subsequently examined the effect of the demethylation system on RNA Pol II levels in the highly methylated region of CpG island in BRD7 promoter. The ChIP‐qPCR results showed that this demethylation system can enhance RNA Pol II recruitment to this region, and the effect is more pronounced when sgRNA2 and sgRNA5 are delivered together (Figure 4G). These results suggest that the LentiCRISPRv2/dCas9‐TET1CD‐sgRNA2&5 demethylation system can transcriptionally activate BRD7 expression by targeting hypermethylated regions of the BRD7 promoter CpG island for demethylation and promoting RNA Pol II recruitment to this region.

### 3.5 Targeted demethylation of the BRD7 promoter inhibits the malignant progression of NPC cells

**FIGURE 5:**
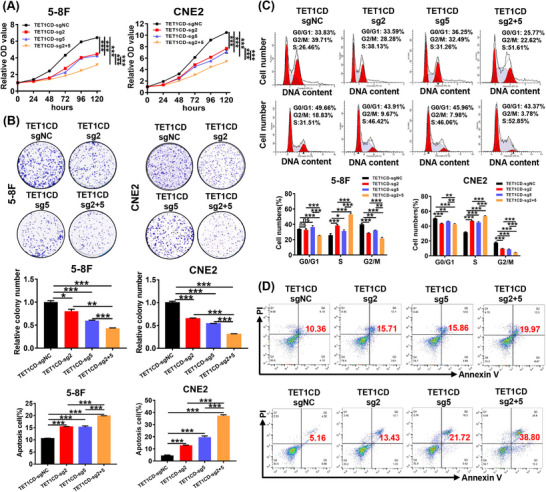
Targeted demethylation of the BRD7 promoter inhibits the malignant progression of nasopharyngeal carcinoma (NPC) cells. (A) CCK‐8 assay (*n* = 5, five replicates per group) was performed to determine the growth of NPC cells stably expressing dCas9‐TET1CD‐sgNC, dCas9‐TET1CD‐sgRNA2, dCas9‐TET1CD‐sgRNA5 and co‐expressing dCas9‐TET1CD‐sgRNA2 and dCas9‐TET1CD‐sgRNA5. (B) Representative and quantified results (*n* = 3, three replicates per group) of the colony formation in 5‐8F and CNE2 cells. (C, D) Flow cytometry was used to detect the effects of the LentiCRISPRv2/dCas9‐TET1CD‐sgRNAs demethylation system on the cell cycle progression (C) and apoptosis (D) of NPC cells 5‐8F and CNE2. In this figure, the data are presented as mean ± standard error of the mean (SEM) (*n* = 3). *p < .05; **p < .01; ***p < .001; ns, no significance.

**FIGURE 6:**
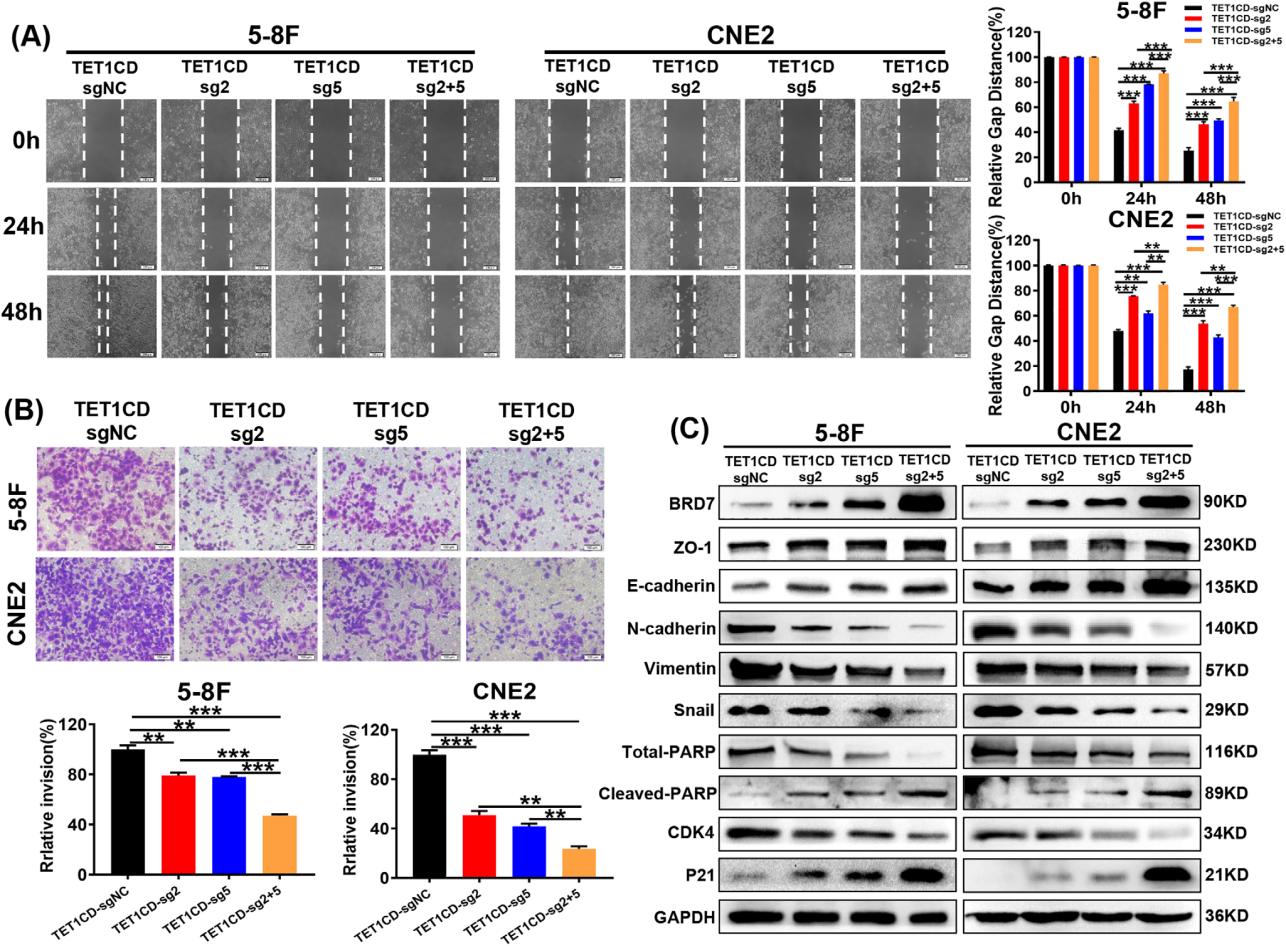
Targeted demethylation of the BRD7 promoter inhibits the migration, invasion and EMT of nasopharyngeal carcinoma (NPC) cells. (A, B) Representative and quantified results (*n* = 3, three replicates per group) of the wound healing (A) and transwell (B) assays in 5‐8F and CNE2 cells that stably express dCas9‐TET1CD‐sgNC, dCas9‐TET1CD‐sgRNA2, dCas9‐TET1CD‐sgRNA5 and co‐expressing dCas9‐TET1CD‐sgRNA2 and dCas9‐TET1CD‐sgRNA5. (C) Western blot was used to analyse the expression changes of EMT, apoptosis and cycle‐related molecules in NPC cells after stable expression of dCas9‐TET1CD‐sgRNA2, dCas9‐TET1CD‐sgRNA5 or their combined expression compared with the control group. In A and B, the data are presented as mean ± standard error of the mean (SEM). **p < .01; ***p < .001.

To determine the potential anti‐tumour effects of BRD7‐targeted demethylation in NPC, we conducted in vitro experiments using NPC cells stably expressing dCas9‐TET1CD‐sgRNA2&5. CCK‐8 and colony formation experiments indicated that sgRNA2 and sgRNA5‐guided dCas9‐TET1CD significantly inhibited the proliferation ability and colony formation ability of NPC cells, with the co‐expression group showing an even more pronounced inhibitory effect (Figure 5A,B), as similar to the results obtained with BRD7 overexpression (Figure S4). The findings from the flow cytometry assay indicated that expression of dCas9‐TET1CD‐sgRNA2&5 caused a blockade of NPC cells in the S phase and promoted apoptosis as compared to the control group, with a more pronounced effect observed in the co‐expression group (Figure 5C,D). Subsequently, we investigated the impact of the demethylation system on the migration ability and invasion ability of NPC cells. Scratch wound healing and Matrigel invasion experiments confirmed that, under the activation of sgRNA2 or sgRNA5‐guided dCas9‐TET1CD system, the migration and invasion of NPC cells were reduced in comparison to the control group, and the combined delivery of sgRNA2 and sgRNA5 exhibited an additive effect, leading to a more significant inhibition (Figure 6A,B). The findings demonstrate the anti‐tumour effects of BRD7‐targeted demethylation in NPC. To further investigate the effects of BRD7 activation mediated by this demethylation system on NPC cell proliferation and epithelial‐mesenchymal transition (EMT) process, we investigated the influence of this demethylation system on the expression of molecules related to the cell cycle, apoptosis and EMT. The findings indicated that the expressions of BRD7, P21, Cleaved‐PARP, ZO‐1 and E‐cadherin were significantly up‐regulated in NPC cells with stable expression of dCas9‐TET1CD‐sgRNA2 or dCas9‐TET1CD‐sgRNA5, while the expressions of CDK4, Total‐PARP, Vimentin, N‐cadherin and Snail were down‐regulated, and the co‐expression group exhibited an additive effect of inhibition or enhancement (Figure 6C). To further enhance the reliability of our mechanism, we also examined the effects of the demethylation system on key downstream molecules of BRD7, specifically BIRC2 and PTEN proteins.^36^, ^37^ The results indicated that stable expression of either dCas9‐TET1CD‐sgRNA2 or sgRNA5 alone could inhibit BIRC2 expression and promote PTEN expression. Moreover, the combined delivery of sgRNA2 and sgRNA5 exhibited a synergistic effect, resulting in a more significant inhibition or enhancement effect (Figure S5). Therefore, these findings suggest that the LentiCRISPRv2/dCas9‐TET1CD‐sgRNAs demethylation system enhances the inhibitory effects on the proliferation, migration and invasion of NPC cells by transcriptionally activating BRD7 expression through targeted demethylation of BRD7 promoter, thus exerting a dramatic tumour inhibitory effect in NPC in vitro.

### 3.6 Restoring BRD7 expression rescues the inhibitory effect of the demethylation system on malignant phenotype of NPC cells

**FIGURE 7:**
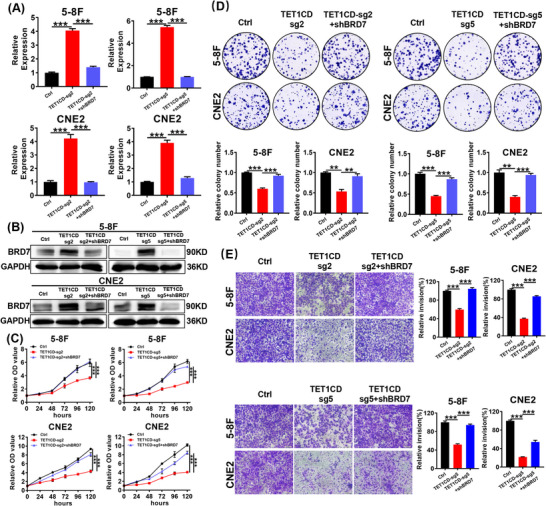
Restoring BRD7 expression rescues the inhibitory effect of the demethylation system on malignant phenotype of nasopharyngeal carcinoma (NPC) cells. (A, B) Reverse transcription‐quantitative polymerase chain reaction (RT‐qPCR) (A) and Western blot (B) assays were used to detect the mRNA and protein expression of BRD7 in each group. (C, D) CCK‐8 assay (*n* = 5, five replicates per group) (C) and colony formation assay (*n* = 3, three replicates per group) (D) were used to detect the effects of stable expression of dCas9‐TET1CD‐sgRNA2, dCas9‐TET1CD‐sgRNA5 and restoring BRD7 expression on the proliferation and colony formation of NPC cells 5‐8F and CNE2. (E) Representative and quantified results (*n* = 3, three replicates per group) of the transwell assays in 5‐8F and CNE2 cells that stably expression of dCas9‐TET1CD‐sgRNA2, dCas9‐TET1CD‐sgRNA5 and restoring BRD7 expression. In A, C–E, the data are presented as mean ± standard error of the mean (SEM) (*n* = 3). **p < .01; ***p < .001.

Considering that dCas9‐TET1CD‐sgRNA2&sgRNA5 can suppress the proliferation, migration and invasion of NPC cells by transcriptionally activating BRD7, we aimed to further clarify the impact of BRD7 reactivation on the suppression of the malignant phenotype mediated by this demethylation system. We further transfected shBRD7 into 5‐8F and CNE2 cells that stably express dCas9‐TET1CD‐sgRNA2&sgRNA5 to restore BRD7 expression, with quality control conducted through RT‐qPCR and Western blot experiments (Figure 7A,B). CCK‐8 and colony formation experiments indicated that, in comparison to the control group, BRD7 transcriptional activation mediated by dCas9‐TET1CD‐sgRNA2&sgRNA5 significantly inhibited the proliferation ability and colony formation ability of NPC cells, while restoring BRD7 expression rescued the inhibitory effect of this demethylation system on NPC cell proliferation and colony formation (Figure 7C,D). Transwell assay findings revealed that, in comparison to the control group, the invasive ability of NPC cells in the groups stably expressing dCas9‐TET1CD‐sgRNA2 or dCas9‐TET1CD‐sgRNA5 was significantly reduced, while restoring BRD7 expression partially attenuated the suppressive impact of this demethylation system on NPC cell invasion (Figure 7E).

### 3.7 The LentiCRISPRv2/dCas9‐TET1CD‐sgRNAs system targets demethylation of the proximal BRD7 promoter to inhibit tumour growth in vivo

**FIGURE 8:**
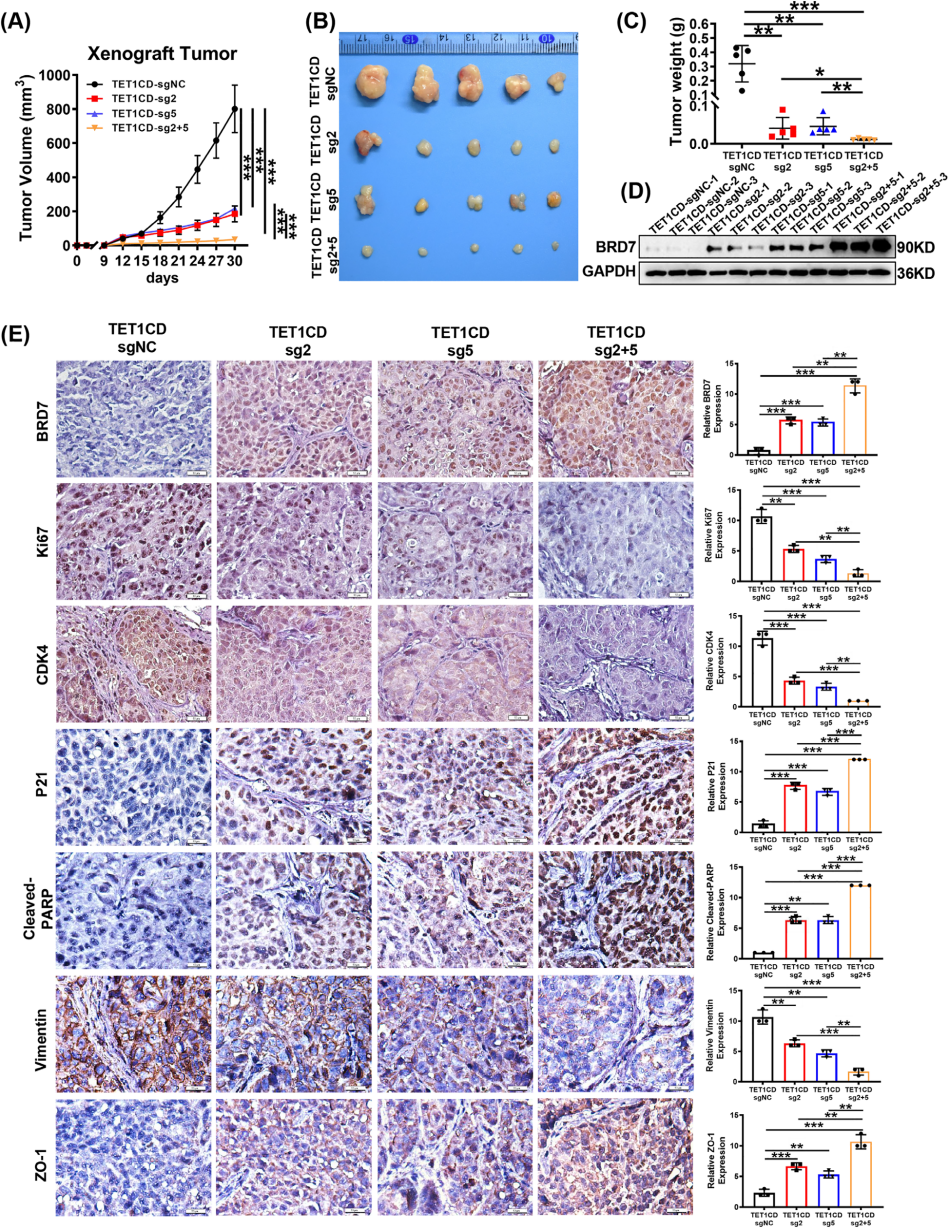
The LentiCRISPRv2/dCas9‐TET1CD‐sgRNAs system targets demethylation of the proximal BRD7 promoter to inhibit tumour growth in vivo. (A) Subcutaneous tumour growth curves of the indicated groups (*n* = 5, five mice for each group). (B) Representative image of the subcutaneous tumour masses derived from each group (*n* = 5). (C) Tumour weight at killing was monitored in the indicated groups (*n* = 5). (D) Western blot experiment was conducted to detect the expression of BRD7 in tumour masses from each group. (E) Representative images and statistical analysis (*n* = 3) of immunohistochemical staining of BRD7, Ki67, CDK4, P21, Cleaved‐PARP, Vimentin and ZO‐1 in vivo tumours in each group. The data are presented as mean ± standard error of the mean (SEM). *p < .05; **p < .01; ***p < .001.

Next, to investigate the long‐term effects of dCas9‐TET1CD‐mediated demethylation on NPC cells tumour growth in vivo, we injected 3 × 10^6^ CNE2 cells stably expressing this demethylation system into the upper armpit of the right forelimb of nude mice to create a subcutaneous tumour model. Tumour size and the weight of the nude mice were recorded every 3 days and all mice were euthanised on day 30 (Figure S6A), and subcutaneous tumours were excised, photographed and weighed. The findings indicated that there were no significant differences in body weight among the groups of nude mice (Figure S6B). The growth curve of the subcutaneous tumours indicated that stable expression of dCas9‐TET1CD‐sgRNA2 and dCas9‐TET1CD‐sgRNA5 markedly suppressed tumour growth in vivo when compared to the control group, and the tumour weights were also considerably reduced relative to those of the control group. Furthermore, the tumour growth inhibition in the co‐expression group of dCas9‐TET1CD‐sgRNA2&sgRNA5 was more pronounced, with lower tumour weights than that of the groups expressing dCas9‐TET1CD‐sgRNA2 or dCas9‐TET1CD‐sgRNA5 alone (Figure 8A–C). Subsequently, we selected tumour tissues from three representative nude mice of each group, and the Western blot results demonstrated that, in comparison to the control group, the expression of BRD7 was elevated in both the stable expression of dCas9‐TET1CD‐sgRNA2 and dCas9‐TET1CD‐sgRNA5 groups. In the combined group, a synergistic effect was observed, with a dramatic increase in BRD7 expression (Figure 8D). The above results confirmed the persistent anti‐tumour effects of the LentiCRISPRv2/dCas9‐TET1CD‐sgRNAs demethylation system by activating BRD7 expression in vivo. Additionally, the results of IHC staining further confirmed these findings (Figure 8E). Meanwhile, we found that compared with the group, there were fewer Ki67‐positive cells in the groups with stable expression of dCas9‐TET1CD‐sgRNA2 or sgRNA5, along with decreased expression of CDK4 and Vimentin, and upregulation of P21, Cleaved‐PARP and ZO‐1. Notably, in the co‐expression group of dCas9‐TET1CD‐sgRNA2 and sgRNA5, the expression of these molecules showed greater changes, aligning with the findings from the in vitro experiments (Figure 8E). Overall, these in vivo data suggest that targeted demethylation of BRD7 is a potential anti‐tumour strategy.

### 3.8 Lentivirus‐mediated BRD7 demethylation system exerts anti‐tumour effect in nude mice

**FIGURE 9:**
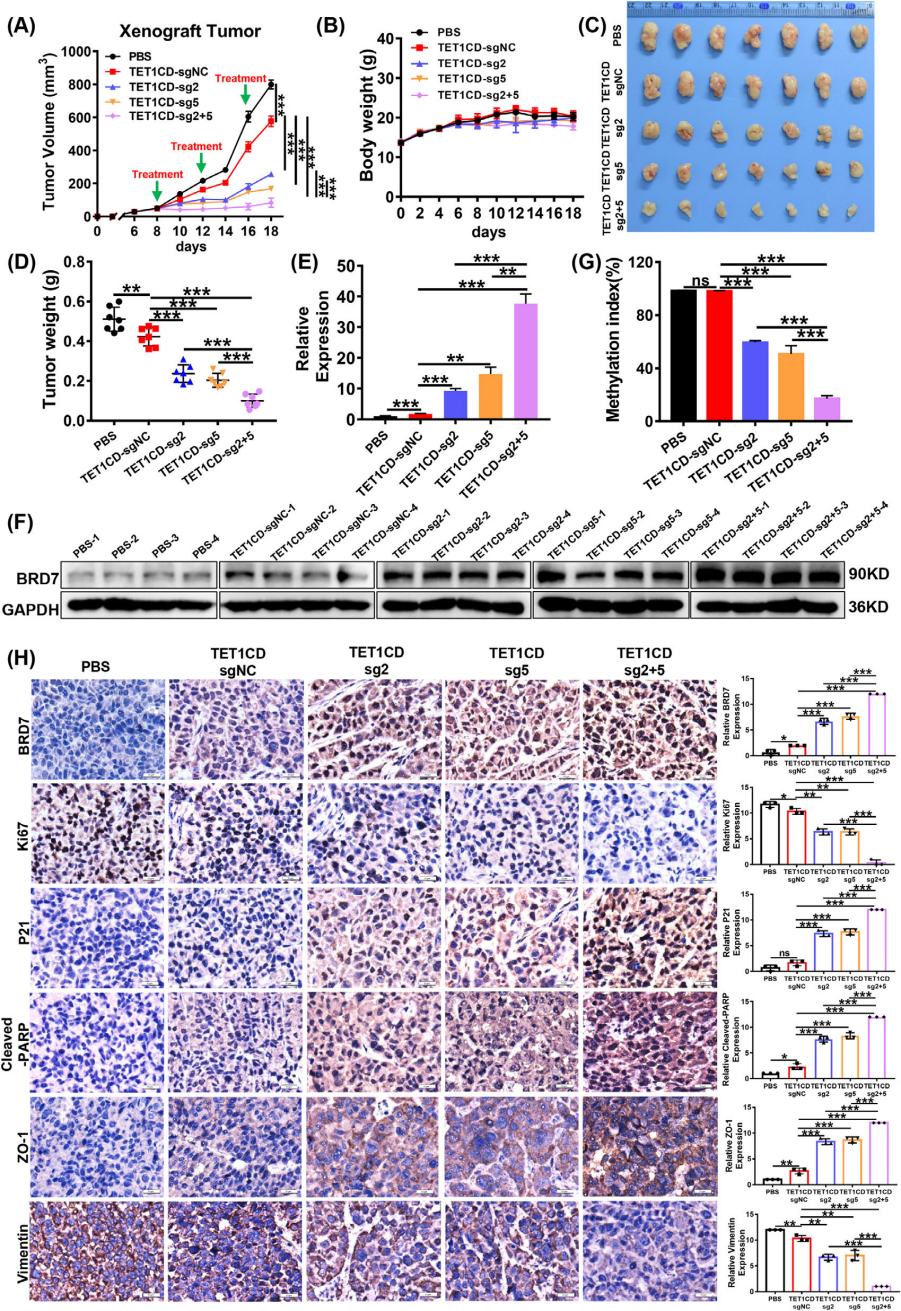
Lentivirus‐mediated BRD7 demethylation system exerts anti‐tumour effect in nude mice. (A) Growth curves of subcutaneous tumours in each group (*n* = 7, seven mice for each group). The arrow represents lentivirus injection treatment. (B) The body weights of nude mice in each group (*n* = 7). (C) Representative image of subcutaneous tumour masses from each group (*n* = 7). (D) The weight of tumour masses in each group (*n* = 7). (E–G) The mRNA (E) and protein (F) expression levels of BRD7 and the methylation level of CpG islands (G) in the promoter region of BRD7 in each group of tumour masses. (H) Representative immunohistochemical staining images and statistical analysis of BRD7, Ki67, P21, Cleaved‐PARP, ZO‐1 and Vimentin in tumour masses of each group in the lentivirus treatment model. In this figure, the data are presented as mean ± standard error of the mean (SEM) (*n* = 3). *p < .05; **p < .01; ***p < .001; ns, no significance.

Although we have demonstrated that BRD7 demethylation system can effectively activate the expression of BRD7 and thereby exerts significant tumour suppressive effects, the success of future clinical applications will largely depend on the efficiency of delivery and safety. Therefore, we next utilised lentivirus with stable expression of LentiCRISPRv2/dCas9‐TET1CD‐sgRNA2&5 targeting demethylated BRD7 to evaluate the anti‐tumour effect in xenograft tumour. Once the tumour volume reached around 40–50 mm^3^, the mice with tumours were randomly allocated into five groups, with seven mice in each group. In the treatment group, 100 µL of lentivirus stably expressing dCas9‐TET1CD‐sgRNA2, dCas9‐TET1CD‐sgRNA5 and combination of them with a concentration of 1 × 10^8^ TU/mL were intratumourally injected, respectively, with a total of three times and once every 4 days. While lentivirus stably expressing dCas9‐TET1CD‐sgNC served as a negative control, and 100 µL PBS‐treatment group acted as a blank control. Tumour volume and body weight were assessed every 2 days, and the nude mice were euthanised when the tumours grew to appropriate sizes in control and blank groups, and tumour tissues were extracted (Figure S7A). As a result, the changes in tumour volume indicated that lentivirus with stable expression of LentiCRISPRv2/dCas9‐TET1CD‐sgRNA2&5 obviously inhibited tumour growth as compared to the control and blank groups, while the combination group demonstrated an even more dramatic tumour suppressive effect shown in Figure 9A. In addition, the tumour volume and weight in the LV‐dCas9‐TET1CD‐sgRNA2 and sgRNA5 groups were significantly reduced, with tumour suppression rates of 68% and 79%, respectively. Among them, the combination group exhibited the most pronounced suppression effect, with a tumour suppression rate of 90%. Additionally, we found that the tumour volume and weight in the negative control group were also reduced compared to the blank control group, indicating that the TET1CD region might contribute to tumour suppression. There was no statistically significant difference in body weight among the groups of nude mice (*p* > .05; Figure 9A–D).

To further validate the impact of the demethylation system on the expression and methylation levels of BRD7 in vivo, we ground a portion of the tumour tissue for RNA and protein extraction. The results from qPCR and Western blot analyses indicated that BRD7 expression was elevated in the LV‐dCas9‐TET1CD‐sgRNA2 and LV‐dCas9‐TET1CD‐sgRNA5 treatment groups compared to the control group. Additionally, the LV‐dCas9‐TET1CD‐sgRNA2 + sgRNA5 treatment group exhibited a synergistic effect, with a more significant increase in BRD7 expression (Figure 9E,F). MSP experiments also confirmed that, in comparison to the control group, the methylation level in BRD7 promoter region decreased in the single treatment groups, and was even more significantly reduced in the combined treatment group. Notably, there was no statistically meaningful difference in methylation levels between LV‐dCas9‐TET1CD‐sgRNANC and PBS groups, suggesting that although TET1CD has a certain tumour suppressive effect in NPC tumour model, this effect is broad‐spectrum and not specifically mediated by demethylation of the BRD7 promoter (Figure 9G). IHC results also confirmed the activation of BRD7 expression. Furthermore, in comparison to the control group, there was a reduction in Ki67‐positive cells, downregulation of the stromal marker molecule Vimentin, upregulation of the apoptosis‐related molecule Cleaved‐PARP, upregulation of the cell cycle‐related molecule P21, and increased expression of the epithelial marker molecule ZO‐1. In the combined treatment group, the expression of these marker molecules showed more pronounced changes (Figure 9H). These results confirm that the lentivirus stably expressing the LentiCRISPRv2‐dCas9‐TET1CD‐sgRNA2&5 demethylation system can effectively infect tumours, leading to the demethylation of the highly methylated CpG island region of the BRD7 promoter, consequently activating BRD7 expression at the transcriptional level and thus exerting anti‐tumour roles in vivo.

Next, we evaluated the safety of delivery of this demethylation system in vivo by preparing histopathological sections of key organs (heart, liver, spleen, lung and kidney) and conducting haematoxylin and eosin (H&E) staining. Under the light microscope, we observed the histopathological changes in these organs, as shown in Figure S7B. In comparison to the control groups, no abnormal structural alterations were noted in these major organ tissues under the microscope in the experimental groups. This indicates that the treatment of lentivirus equipped with these demethylation systems had no obvious toxic effects on the major organs of nude mice, and to a certain extent, confirmed the safety of lentivirus‐mediated demethylation system therapy in vivo. To further assess the safety of the lentivirus‐mediated demethylation system in tumour therapy, we conducted acute toxicology experiments, and the results are shown in Figure S8. The blood parameters of the mice (including white blood cell and red blood cell counts, platelet counts and haemoglobin concentration) showed no marked changes, indicating no haematological abnormalities. Additionally, we found that the liver function indicators alanine aminotransferase (ALT) and aspartate aminotransferase (AST) and kidney function indicators creatinine (CREA) and blood urea nitrogen (BUN) of the mice were all within the normal range, suggesting that our lentiviral delivery system did not cause significant hepatotoxicity or nephrotoxicity. These results further validate the in vivo safety and good biocompatibility of the lentivirus‐mediated BRD7 demethylation system.

### 3.9 The decreased expression of BRD7 is associated with the hypermethylation of the BRD7 promoter in biopsy tissues of NPC

**FIGURE 10:**
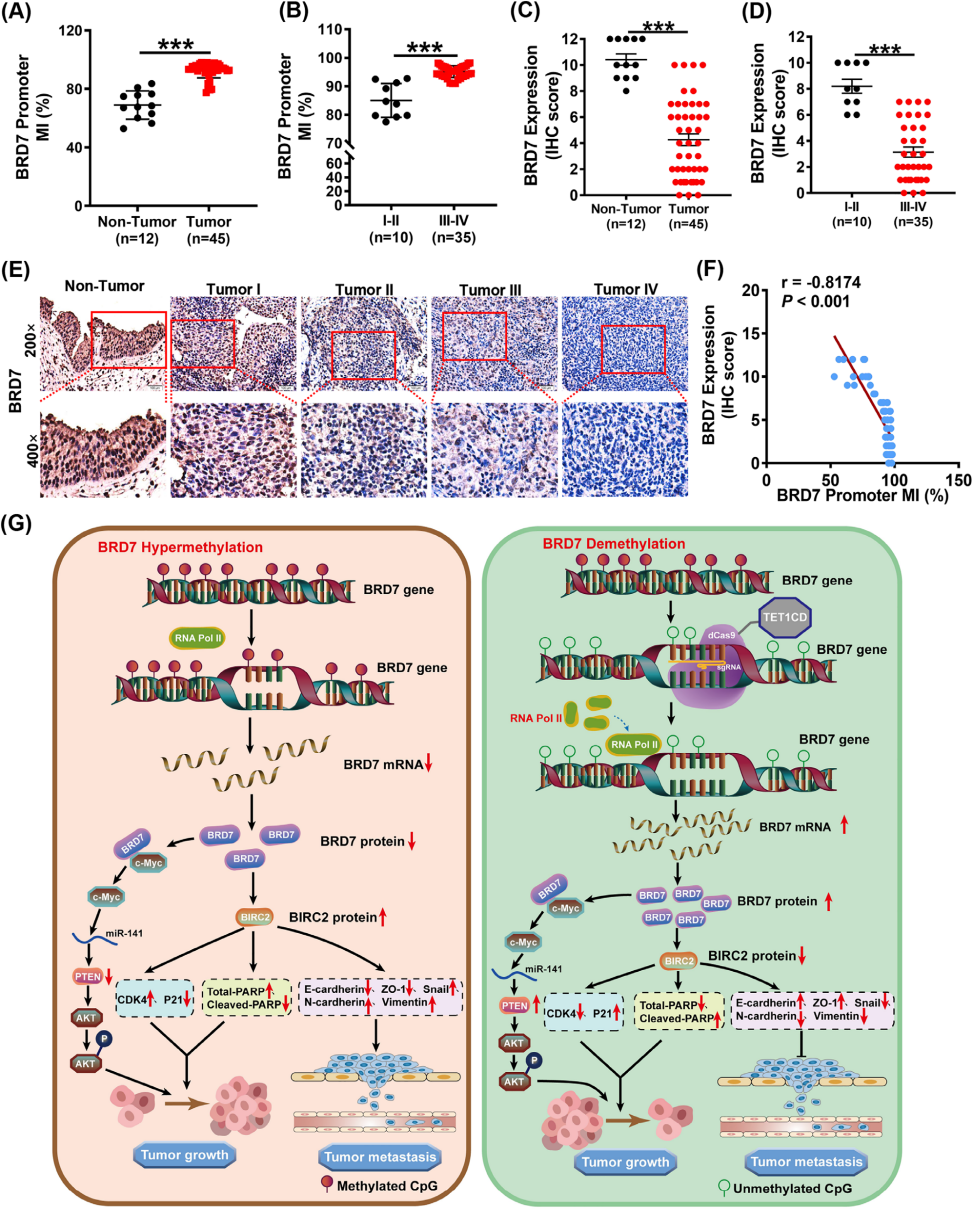
The decreased expression of BRD7 is associated with the hypermethylation of the BRD7 promoter in biopsy tissues of nasopharyngeal carcinoma (NPC). (A, B) Statistical quantification charts of qMSP experiments to detect the methylation levels of the BRD7 promoter region CpG islands in non‐cancerous nasopharyngeal paraffin tissues (*n* = 12) and nasopharyngeal carcinoma paraffin tissues (*n* = 45) at different clinical TNM stages. (C–E) Statistical quantitative charts (C, D) and representative images (E) of BRD7 expression in non‐cancerous nasopharyngeal paraffin tissues (*n* = 12) and nasopharyngeal carcinoma paraffin tissues (*n* = 45) at different clinical TNM stages detected by immunohistochemical (IHC) assay. The tissue sections used here are the same as those in A and B, and they are all derived from the same patient's paraffin‐embedded tissue. (F) Correlation analysis of BRD7 expression and methylation level in the CpG island region of its promoter region in paraffin tissues (*n* = 57). (G) Schematic diagram of the molecular mechanism by which the LentiCRISPRv2/dCas9‐TET1CD‐sgRNAs demethylation system targets the BRD7 promoter region for demethylation and transcriptional activation of BRD7 expression, thereby inhibiting the malignant progression of NPC.

To gain a deeper insight into the correlation and clinical significance between BRD7 expression and promoter methylation levels in NPC, we detected the methylation status of CpG island region of BRD7 promoter and BRD7 expression in 12 non‐cancerous NP patients and 45 NPC patients at different stages using qMSP and IHC, respectively. The findings indicated that, compared to non‐cancerous NP tissues, BRD7 expression levels were lower in NPC tissues, and the methylation level in CpG island region of BRD7 promoter was higher. It was also observed that the BRD7 expression was notably reduced in advanced clinical stages (III–IV) compared to early stages (I–II), while the methylation level in CpG island region of BRD7 promoter region was significantly higher in clinical stages III and IV compared to stages I and II (Figure 10A–E). A negative correlation was identified between BRD7 expression and the methylation levels of its promoter region CpG island in these tissues (*r* = −.8174; *p* < .001; Figure 10F). Additional analysis was performed on the correlation between BRD7 expression, BRD7 promoter region CpG island methylation and their combined association with the clinical pathological features of NPC. The results indicated that BRD7 expression was not significantly correlated with NPC patients age or gender; however, it was significantly negatively correlated with clinical stage. Similarly, the methylation levels of the BRD7 promoter CpG island were not significantly correlated with the age and gender of the patients; however, they were significantly positively correlated with clinical stage. Additionally, we found that hypomethylation and hypermethylation of the BRD7 promoter were respectively linked to the increase and decrease of BRD7 expression levels in NPC specimens. These results suggest that hypermethylation of the CpG island in the BRD7 gene promoter is a key factor leading to the low expression of BRD7 (Table 1). Overall, targeting the methylation region of CpG island within BRD7 promoter represents a promising clinical diagnostic and therapeutic strategy for NPC.

**TABLE 1:** Association between the expression of BRD7, BRD7 methylation index and nasopharyngeal carcinoma (NPC) clinical pathological features (*n* = 45).

	BRD7 expression			BRD7 methylation index			BRD7/BRD7 methylation index		
Variables features	Low	High	*p*	Low	High	*p*	L–H	H–L	*p*
Gender									
Male (*n* = 31)	24 (77%)	7 (23%)	.3564	16 (52%)	15 (48%)	.9202	15 (48%)	7 (23%)	.5658
Female (*n* = 14)	9 (64%)	5 (36%)		7 (50%)	7 (50%)		7 (50%)	5 (36%)	
Age									
≤53 (*n* = 23)	18 (78%)	5 (22%)	.4447	11 (48%)	12 (52%)	.6522	12 (52%)	5 (22%)	.4729
>53 (*n* = 22)	15 (68%)	7 (32%)		12 (55%)	10 (45%)		10 (45%)	7 (32%)	
Clinical stages									
I–II (*n* = 10)	2 (20%)	8 (80%)	<.001^***^	10 (100%)	0 (0%)	.0005^***^	0 (0%)	8 (80%)	<.001^***^
III–IV (*n* = 35)	31 (89%)	4 (11%)		13 (37%)	22 (63%)		22 (63%)	4 (11%)	

The following abbreviations were used: H, high expression; L, low expression. Statistical analysis was performed using the chi‐squared test.

*** *p* < .001.

## 4 DISCUSSION

Our preliminary research has confirmed that BRD7 is down‐regulated in NPC and functions as a tumour suppressor gene.^4^, ^5^, ^6^, ^7^ The downregulation of its expression is a significant mechanism that contributes to the occurrence and progression of NPC. Therefore, targeting BRD7 activation may represent a crucial molecular strategy for treating NPC. In previous studies, it was found that BRD7 was lowly expressed at both the mRNA and protein levels, and it was confirmed that BRD7 was negatively regulated by c‐Myc at the transcriptional level,^9^ but this is insufficient to explain the mechanism of the down‐regulated expression of BRD7 mRNA in NPC. Previous studies have indicated that DNA methylation plays a significant role in the downregulation of mRNA expression, with the low levels of many tumour suppressor genes' mRNAs attributed to the hypermethylation of the CpG islands in their promoter regions, such as p16, Rb, PTEN, ZNF154 and BRCA1.^10^, ^11^, ^12^, ^15^, ^16^, ^17^, ^18^, ^19^, ^20^ In our study, we predicted through bioinformatics that there is hypermethylation modification in the CpG island region of the BRD7 promoter. Moreover, the MSP experiments confirmed that there was hypermethylation occurred in the CpG island in the BRD7 promoter in NPC cell lines and tissues, which is negatively correlated with BRD7 expression. This confirms that hypermethylation of the CpG island in BRD7 promoter is a key mechanism responsible for the downregulation of BRD7 expression in NPC.

Although DNA methylation is a highly stable silencing marker, it is a reversible regulatory process, and reducing its hypermethylation modification may restore gene expression.^21^, ^22^ Therefore, targeting BRD7 demethylation can serve as an important molecular strategy for activating BRD7 and subsequent tumour treatment. Decitabine is a reported effective inhibitor of DNA methyltransferases.^23^, ^24^, ^25^, ^26^ In this study, we treated NPC cell lines 5‐8F and CNE2 with decitabine and confirmed that this treatment significantly reduced methylation levels of the CpG islands in BRD7 promoter in NPC cells, thereby restoring BRD7 expression. However, as a broad‐spectrum demethylating drug, it cannot precisely target the demethylation of a certain gene, leading to widespread off‐target effects and resulting in various toxic side effects.^27^ CRISPR/dCas9 is a system developed by modifying and upgrading the CRISPR/Cas9 gene editing system for the targeted regulation of genomic transcription. It takes advantage of the precision and targeting of the CRISPR/Cas9 system to fuse the dCas9 protein with various effector proteins' catalytic domains, such as the DNA demethylase TET1, the transcriptional activator VP64, and the transcriptional repressor KRAB, and so forth, thereby targeting these functional domains to specific regions and achieving precise regulation of genes.^28^, ^29^, ^30^ Therefore, to activate BRD7 expression through specific demethylation, we constructed a BRD7‐targeted demethylation system. In simple terms, we replaced the Cas9 component in LentiCRISPRv2 plasmid with dCas9‐TET1CD and designed five sgRNAs based on the methylation regions of the CpG island in the BRD7 promoter, which were then inserted into the demethylation system, respectively, completing the construction of the LentiCRISPRv2/dCas9‐TET1CD‐sgRNAs demethylation system. Consequently, this demethylation system is capable of continuously expressing the sgRNA and dCas9‐TET1CD fusion protein, enabling targeted and sustained demethylation and transcriptional activation of the target genes. Our research findings indicated that the five sgRNAs (sgRNA1–5) guided dCas9‐TET1CD demethylation systems could activate BRD7 expression to varying degrees, thereby inhibiting the proliferation and colony formation of NPC cells, with sgRNA2 and sgRNA5 showing the most significant effects. To further confirm the effectiveness of this system and its potential application in tumour therapy, we constructed NPC cell lines stably transfected with dCas9‐TET1CD‐sgRNA2 and dCas9‐TET1CD‐sgRNA5. A series of in vitro experiments demonstrated that the demethylation systems guided by sgRNA2 and sgRNA5 could promote transcriptional activation and BRD7 expression by reducing its methylation, inhibiting the proliferation, migration, invasion of NPC cells and tumour growth in vivo. Moreover, the combined use of both sgRNAs exhibited more pronounced effects in demethylation, transcriptional activation and tumour suppression. Similarly, He et al. developed a targeted demethylation system utilising CRISPR/dCas9 technology, which confirmed that dCas9‐TET1CD induced demethylation of the ZNF154 promoter could activate ZNF154 expression, thereby inhibiting the proliferation and migration of ESCC cells.^19^ Choudhury et al. demonstrated that under the guidance of sgRNA, the dCas9‐TET1CD fusion protein could demethylate the BRCA1 promoter, leading to the upregulation of transcription of this gene and then inhibiting the proliferation of breast cancer cells.^32^ The above mentioned studies based on CRISPR/dCas9‐TET1CD demethylation systems provide evidence for the effectiveness of our system. In addition, we found that the sgRNA binding region does not completely correspond to the regions where demethylation occurs, indicating the presence of some adjacent effects. This suggests that the position of the sgRNA binding site relative to the target CpG sites may affect the degree of demethylation. In fact, a similar situation was also observed in the study by Choudhury et al.,^32^ suggesting that solely relying on sgRNA design based on highly methylated sites may not accurately reflect its demethylation effects, and its effectiveness still requires experimental validation.

Although we have confirmed through a series of in vitro experiments that the use of this demethylation system can effectively activate BRD7 expression, thereby exerting a significant tumour suppressor effect, the success of future clinical applications will largely depend on the safety and efficacy of this system. Therefore, we investigated the therapeutic effects of this system on NPC. The demethylation system constructed in this study is a lentiviral vector, as lentiviruses possess strong transgenic delivery capabilities and can achieve long‐term stable expression in vivo.^41^, ^42^ We encapsulated the demethylation system using lentivirus and established a xenograft tumour model using NPC cell line. Once the tumours attained a size of 40–50 mm^3^, the lentivirus loaded with dCas9‐TET1CD‐sgRNA2&5 was injected into the grafted tumours by intratumoural multi‐point injection. It was confirmed that dCas9‐TET1CD‐sgRNA2&5 could inhibit tumour growth in vivo by targeting BRD7 for transcriptional activation, demonstrating good anti‐tumour effects without exhibiting in vivo toxic side effects. In addition, comprehensive blood biochemical and haematological analyses conducted on the treated group of animals showed that their liver and kidney functions, as well as haematological parameters, were not significantly affected, further demonstrating the good safety and biocompatibility of our lentivirus‐based demethylation system delivery strategy in vivo. This indicates that the delivery of a lentivirus‐mediated demethylation system has potential applications in the future treatment of NPC. However, the lentiviral delivery methods still face challenges. Once lentiviral vector enters the body, it may be recognised by host pattern recognition receptors, triggering an innate immune response.^43^ Additionally, they can integrate into the host genome, posing a risk of insertional mutagenesis and a certain potential for tumourigenesis. Therefore, its safety and immunogenicity issues still need further validation.^44^ Currently, widely studied viral delivery systems include not only lentiviruses but also adenoviruses, adeno‐associated viruses (AAVs), and so forth.^45^ Among them, AAV is a non‐pathogenic virus that offers benefits such as low immunogenicity, non‐integration and good stability in vivo. However, its limited packaging capacity (∼4.7 kb) restricts its carrying ability.^46^ Additionally, recent studies have shown that nanoparticle delivery systems are gaining increasing attention for their roles in gene expression or regulatory systems and drug delivery.^47^, ^48^ These systems have the potential to overcome many limitations of viral vectors, particularly in terms of safety, large packaging capacity, reduced off‐target effects, low immunogenicity and in vivo applications. Additionally, they can achieve systemic delivery through intravenous injection, breaking the limitations of local injection.^49^ Therefore, in subsequent studies, efforts can be made to optimise the delivery system to bring it closer to clinical application. However, whether the effectiveness and safety of these delivery methods in mediating the demethylation system of the target gene are better still need to be confirmed through further investigation. Meanwhile, we found that TET1CD may also exhibit some anti‐tumour effects within this system; however, due to its lack of targeting capability, this effect may be very limited. In the methylation detection of tumour masses in the xenograft mouse model, it was also found that TET1CD had a very limited demethylation effect on the CpG island region of the BRD7 promoter, indicating that the anti‐tumour effects of TET1CD in NPC are not due to specific targeting of BRD7 for demethylation. The specific mechanisms through which TET1CD exerts its effects still need to be further explored.

Previous studies have shown that BRD7 inhibits the proliferation of NPC cells by regulating signalling pathways such as PI3K/AKT, ras/MEK/ERK, Rb/E2F and Wnt/β‐catenin.^4^, ^5^, ^6^, ^7^ This suggests that this demethylation system may inhibit tumour progression by transcriptionally activating BRD7 and thus regulating these pathways. Additionally, recent research indicates that BRD7 can downregulate PD‐L1 expression by inhibiting the PI3K/AKT/mTOR/STAT3 signalling pathway, thereby enhancing the cytotoxic function of CD8^+^ T cells and suppressing immune evasion in NPC, which in turn inhibits tumour growth.^50^ Furthermore, literature has also indicated that BRD7 is involved in regulating glucose metabolism and insulin signalling pathways, thereby influencing processes such as obesity and embryonic development.^51^ However, whether our demethylation system affects tumour progression by regulating the immune microenvironment and metabolic pathways still requires further investigation and clarification in our subsequent studies.

## 5 CONCLUSIONS

In summary, the hypermethylation of the CpG island in BRD7 promoter mediates the transcriptional inactivation of BRD7 in NPC. The demethylation system constructed based on CRISPR/dCas9 and lentiviral delivery technology, LentiCRISPRv2/dCas9‐TET1CD‐sgRNA (sgRNA1‐5), can activate BRD7 expression to varying degrees and inhibit the proliferation of tumour cells, with sgRNA2 and sgRNA5 showing the most significant effects. Furthermore, it was confirmed that the demethylation system guided by sgRNA2&5 could promote transcriptional activation and BRD7 expression through reducing its methylation, thereby inhibiting the proliferation, migration, invasion and tumour growth in vivo of NPC cells, with a more pronounced effect when both are used together. Importantly, we demonstrated that the lentiviral particles encapsulating this system can effectively activate BRD7 expression and exert anti‐tumour effects. Additionally, it has been confirmed in clinical specimens of NPC that there are hypermethylation modifications in the CpG island region of BRD7 promoter, which were negatively correlated with BRD7 expression. In conclusion, these research findings indicate that the hypermethylation modification of BRD7 promoter region serves as a critical molecular mechanism leading to BRD7 transcriptional inactivation and the malignant progression of NPC. The lentiviral delivery system targeting BRD7 demethylation holds promise as a promising molecular strategy for NPC therapy (Figure 10G).

## AUTHOR CONTRIBUTIONS

Ming Zhou and Jianxia Wei conceived and designed this study, wrote and revised the manuscript. Jianxia Wei mainly performed the experiment and interpreted the data. Mengna Li was involved in providing guidance on experimental techniques. Yumei Duan performed the nude mouse sections of H&E. Changning Xue, Lemei Zheng, Qingqing Wei, Zubing Wu, Huizhen Xin, Ting Zeng and Hongyu Deng helped solution preparation and reagents ordering. Songqing Fan contributed to collection and grading of clinical samples. Wei Xiong and Zhaoyang Zeng provided important comments and opinions for the manuscript. All the authors read and approved the final manuscript.

## CONFLICT OF INTEREST STATEMENT

The authors declare no conflicts of interest.

## ETHICS STATEMENT

This study was approved by Ethics Review Committees/Institutional Review Boards of Central South University (Changsha, China). And all the animal experiments in this study were approved by the Institutional Animal Care and Use Committee (IACUC) of Central South University (Changsha, China). Clinical samples of NPC and NPE samples were all collected from the Second Xiangya Hospital of Central South University. All patients involved in this article signed informed consent.

## CONSENT FOR PUBLICATION

Not applicable.

## Supporting information



Supporting Information

## Data Availability

The data that support the findings of this study are available in this published article and its supporting information file.
