# Supplementary material for: Targeted demethylation of the BRD7 promoter based on CRISPR/dCas9 system inhibits the malignant progression of nasopharyngeal carcinoma
Source: Clin Transl Med. 2026 Jan 9;16(1):e70583. doi: 10.1002/ctm2.70583 (PMC12784210; doi:10.1002/ctm2.70583)
Supplement: Supplementary file 1 — Supporting Information [file CTM2-16-e70583-s001.pdf]

Targeted demethylation of the BRD7 promoter based on CRISPR/dCas9 system inhibits the malignant progression of nasopharyngeal carcinoma

Jianxia Wei[1,2,3], Yumei Duan[2,4], Changning Xue[1,2,3], Lemei Zheng[1,2,3], Qingqing Wei[1,2,3], Zubing Wu[1,2,3], Huizhen Xin[1,2,3], Ting Zeng[1,2,3], Hongyu Deng[1], Songqing Fan[5], Wei Xiong[1,2,3], Zhaoyang Zeng[1,2,3], Mengna Li[1,2,3*], Ming Zhou[1,2,3*]

[1] NHC Key Laboratory of Carcinogenesis, Hunan Key Laboratory of Oncotarget Gene, Hunan Cancer Hospital and the Affiliated Cancer Hospital of Xiangya School of Medicine, Central South University, Changsha, China;

[2] Cancer Research Institute and Xiangya School of Basic Medical Sciences, Central South University, Changsha, China;

[3] The Key Laboratory of Carcinogenesis and Cancer Invasion of the Chinese Ministry of Education, Central South University, Changsha, China;

[4] Department of Pathology, the Xiangya Hospital, Central South University, Changsha, China;

[5] Department of Pathology, the Second Xiangya Hospital, Central South University, Changsha, China.

***Corresponding author:**

Ming Zhou, PhD, Cancer Research Institute, Central South University, 110 Xiangya Road, Changsha, Hunan 410078, China. E-mail: zhouming2001@163.com

Mengna Li, Hunan Key Laboratory of Oncotarget Gene, Hunan Cancer Hospital and the Affiliated Cancer Hospital of Xiangya School of Medicine, Central South University, 283 Tongzipo Road, Changsha, Hunan 410078, China. E-mail: limengna@hnca.org.cn

Supplementary information

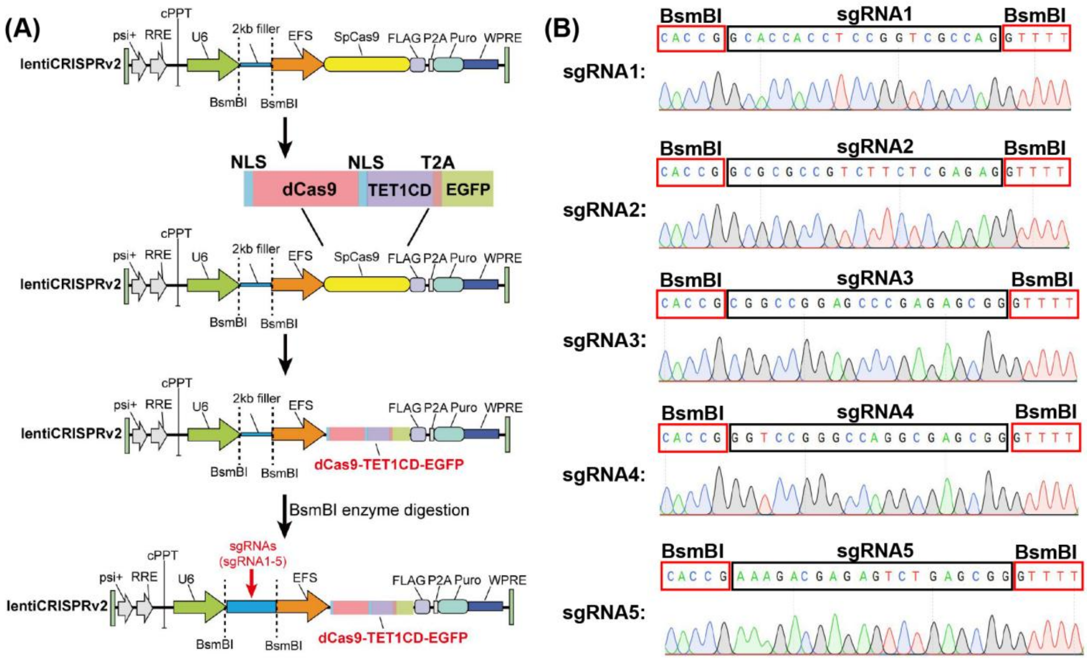

Figure S1 Construction of the LentiCRISPRv2/dCas9-TET1CD-sgRNAs demethylation system. (**A**) The construction process of the LentiCRISPRv2/dCas9-TET1CD-sgRNAs demethylation system. (**B**) Sanger sequencing results of five sgRNAs targeting the hypermethylated region of the CpG island in the promoter region of BRD7.

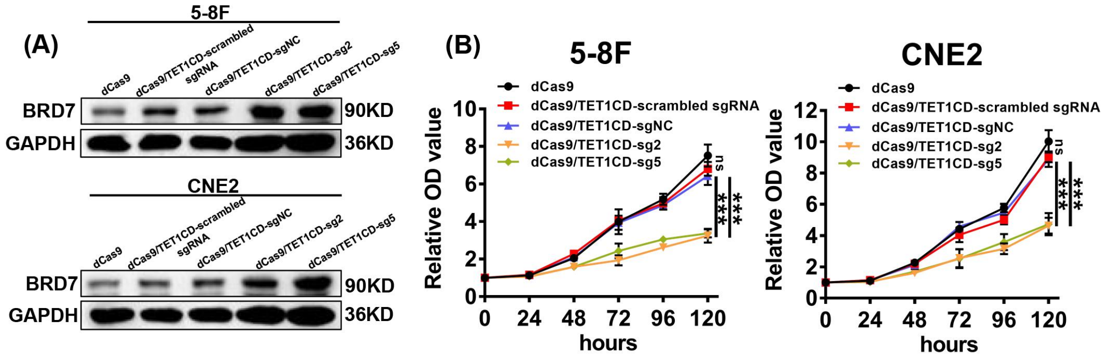

Figure S2 The LentiCRISPRv2/dCas9-TET1CD-sgRNAs demethylation system specifically activates BRD7 and inhibits the proliferation of NPC cells. (**A, B**) Western Blot (**A**) and CCK-8 (**B**) assays were conducted to assess the effects of expressing dCas9, dCas9/TET1CD-scrambled sgRNA, dCas9/TET1CD-sgNC, dCas9/TET1CD-sg2, and dCas9/TET1CD-sg5 on the expression of BRD7 protein (**A**) and the proliferation ability (**B**) of NPC cells.

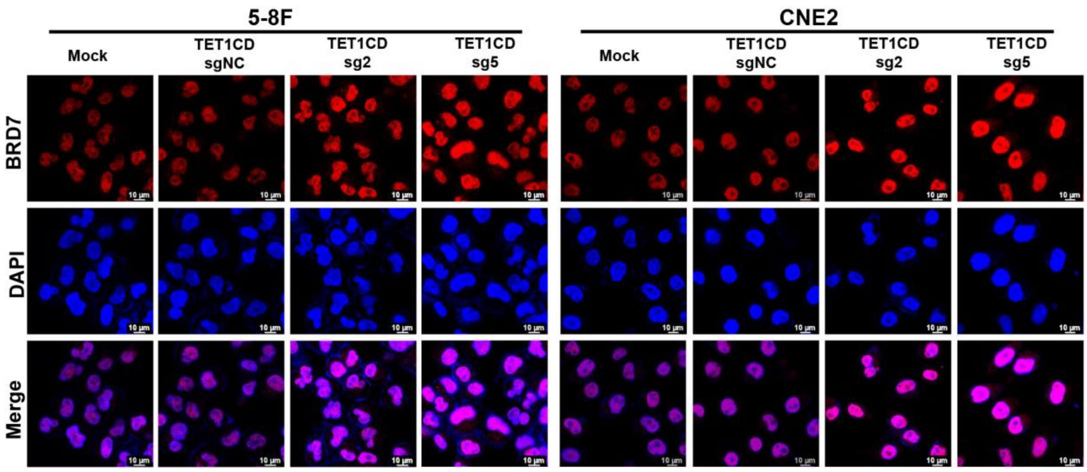

Figure S3 The results of the subcellular localization of BRD7 before and after the demethylation system treatment. Using a laser confocal microscope, the subcellular localization and distribution of BRD7 in untreated NPC cells, as well as in the groups of NPC cells that stably express dCas9-TET1CD-sgNC, dCas9-TET1CD-sg2, and dCas9-TET1CD-sg5, were observed. BRD7 was stained by anti-BRD7 (red) antibodies. Scale bar, 10μm.

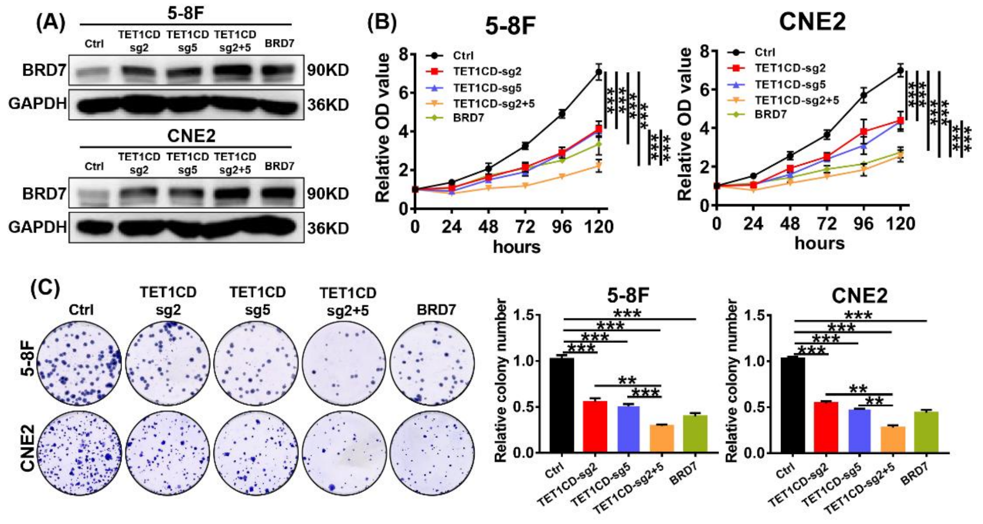

Figure S4 Comparing our demethylation system with the direct overexpression of BRD7. (**A**) BRD7 protein levels in NPC cells that stably express the demethylation system and overexpress BRD7. (**B, C**) CCK-8 assay (**B**) (n = 5, five replicates per group) and colony formation assay (**C**) (n = 3, three replicates per group) were performed to determine the growth and colony formation ability of NPC cells stably expressing dCas9-TET1CD-sgNC, dCas9-TET1CD-sgRNA2, dCas9-TET1CD-sgRNA5, co-expressing dCas9-TET1CD-sgRNA2 and dCas9-TET1CD-sgRNA5 and overexpress BRD7.

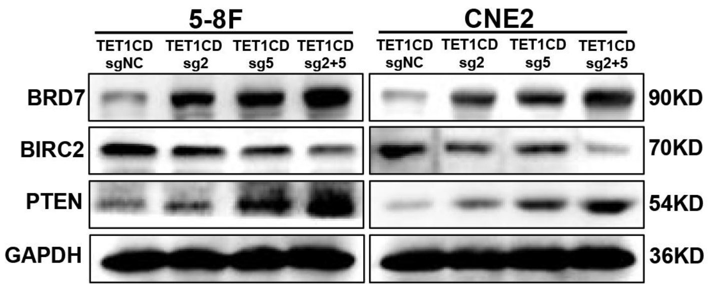

Figure S5 The influence of the demethylation system on downstream molecules of BRD7. Western Blot experiments were conducted to examine the effects of the dCas9-TET1CD-sgRNAs demethylation system on the expression of the downstream target proteins BIRC2 and PTEN of BRD7.

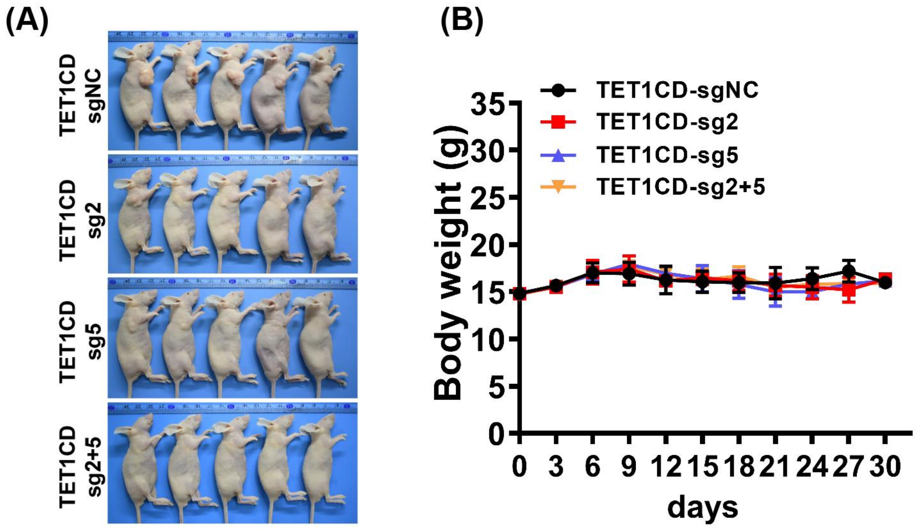

Figure S6 Images and body weight of tumor-bearing nude mice in the NPC transplantation tumor model. (**A**) Images of tumor-bearing nude mice in each group after sacrificed. (**B**) The body weight of tumor-bearing nude mice during the construction of the NPC transplantation tumor model.

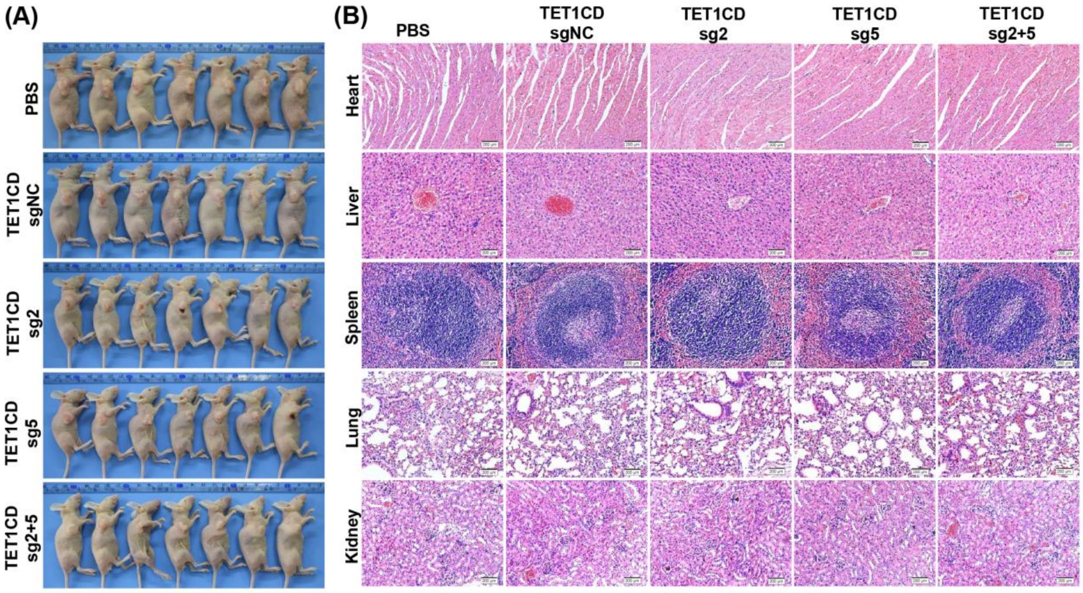

Figure S7 Images of tumor-bearing mice and organ safety detection in the lentivirus treatment model. (**A**) Images of tumor-bearing nude mice in each group after sacrificed in the NPC transplantation tumor model treated with lentivirus. (**B**) Representative images of H&E staining to detect the histopathological changes of the heart, liver, spleen, lung and kidney of nude mice in each group of the lentivirus treatment model.

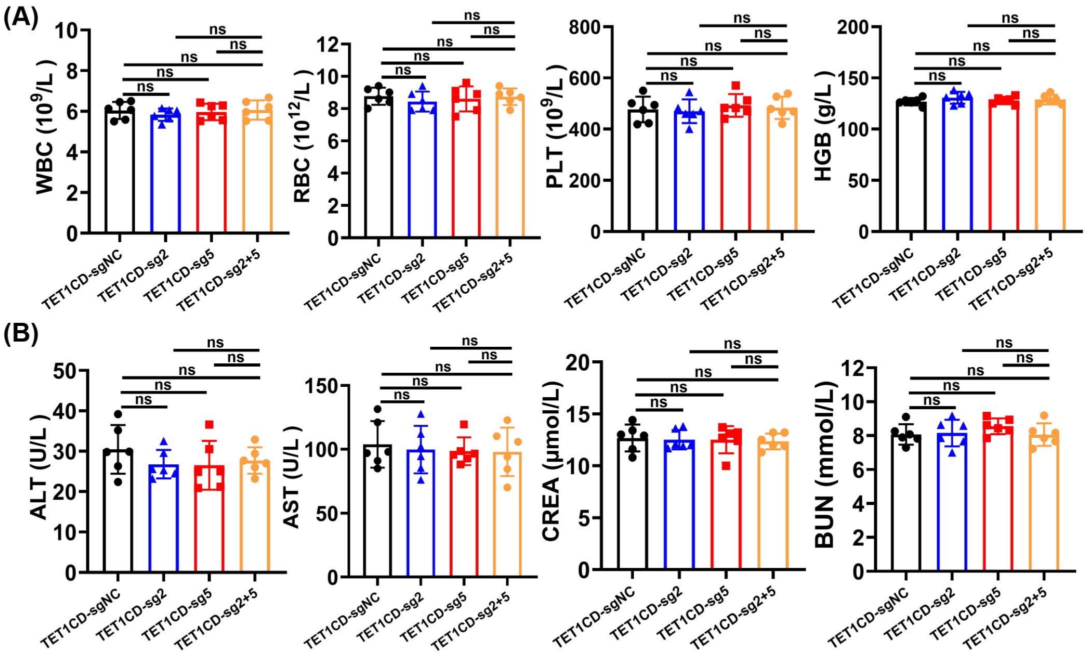

Figure S8 The LentiCRISPRv2/dCas9-TET1CD-sgRNAs demethylation system has no side cytotoxicity in vivo. (**A**) To detect the effects of the lentivirus-mediated dCas9-TET1CD-sgRNAs demethylation system treatment on the blood parameters (such as white blood cell count (WBC) and red blood cell count (RBC), platelet count (PLT), and hemoglobin concentration (HGB)) of mice. (**B**) After treating the mice with the lentivirus-mediated dCas9-TET1CD-sgRNAs demethylation system, the levels of alanine aminotransferase (ALT), aspartate aminotransferase (AST), creatinine (CREA) and blood urea nitrogen (BUN) in their serum were measured.

Table S1. The PCR primers sequences used for methylation-specific PCR and quantitative methylation specific-PCR.

Name	Sequences（5'-3'）	Primer Length (bp)	Genomic locations relative to the sgRNAs	CpG sites measured
Methylation-Forward	AAAGACGAGAGTTTGAGCGGTGGA	24	-379/-356	The upstream region (-379/-1) of the BRD7 transcription initiation site
Methylation-Reverse	ATCCGACCGAACCCCGATACCC	22	-1/-22	
Unmethylation-Forward	AGATGAGAGTTTGAGTGGTGGATTTTG T	28	-377/-350	The upstream region (-377/-1) of the BRD7 transcription initiation site
Unmethylation-Reverse	ATCCAACCAAACCCCAATACC	21	-1/-21	

The translation start site is position +1 and the rest of the sequence is numbered relative to it.

Table S2. The sgRNAs sequences that specifically target the hypermethylated region of the BRD7 promoter CpG island.

Name	Sequences（5’-3’）	On-Target Efficacy Score	Orientation	Target region of sgRNA	Primer Length (bp)
sgRNA1-Forward	CACCGGCACCACCTCCGGTCGCCAG	1.202	sense	-307~-288	25
sgRNA1-Reverse	AAACCTGGCGACCGGAGGTGGTGCC				25
sgRNA2-Forward	CACCGGCGCGCCGTCTTCTCGAGAG	1.139	sense	-175~-156	25
sgRNA2-Reverse	AAACCTCTCGAGAAGACGGCGCGCC				25
sgRNA3-Forward	CACCGCGGCCGGAGCCCGAGAGCGG	1.074	antisense	-182~-201	25
sgRNA3-Reverse	AAACCCGCTCTCGGGCTCCGGCCGC				25
sgRNA4-Forward	CACCGGGTCCGGGCCAGGCGAGCGG	0.8788	antisense	-62~-81	25
sgRNA4-Reverse	AAACCCGCTCGCCTGGCCCGGACCC				25
sgRNA5-Forward	CACCGAAAGACGAGAGTCTGAGCGG	0.8064	sense	-379~-360	25
sgRNA5-Reverse	AAACCCGCTCAGACTCTCGTCTTTC				25

Table S3. The primers sequences for ChIP-qPCR assay.

Name	Sequences（5’-3’）	Primer Length (bp)
ChIP-P1-Forward	TGGAGACGGAGGGAGAGAC	19
ChIP-P1-Reverse	GCAGAGGCGGGAAAGAAGG	19
ChIP-P2-Forward	TTCTTTCCCGCCTCTGCG	18
ChIP-P2-Reverse	GTCCGACCGGGCCCC	15
